# COVID-19 Risk Stratification and Mortality Prediction in Hospitalized Indian Patients: Harnessing clinical data for public health benefits

**DOI:** 10.1371/journal.pone.0264785

**Published:** 2022-03-17

**Authors:** Shanmukh Alle, Akshay Kanakan, Samreen Siddiqui, Akshit Garg, Akshaya Karthikeyan, Priyanka Mehta, Neha Mishra, Partha Chattopadhyay, Priti Devi, Swati Waghdhare, Akansha Tyagi, Bansidhar Tarai, Pranjal Pratim Hazarik, Poonam Das, Sandeep Budhiraja, Vivek Nangia, Arun Dewan, Ramanathan Sethuraman, C. Subramanian, Mashrin Srivastava, Avinash Chakravarthi, Johnny Jacob, Madhuri Namagiri, Varma Konala, Debasish Dash, Tavpritesh Sethi, Sujeet Jha, Anurag Agrawal, Rajesh Pandey, P. K. Vinod, U. Deva Priyakumar

**Affiliations:** 1 Center for Computational Natural Sciences and Bioinformatics, International Institute of Information Technology, Hyderabad, Telangana, India; 2 INtegrative GENomics of HOst-PathogEn (INGEN-HOPE) laboratory, CSIR-Institute of Genomics and Integrative Biology (CSIR-IGIB), Delhi, India; 3 Max Super Speciality Hospital (A Unit of Devki Devi Foundation), Max Healthcare, Delhi, India; 4 Intel Technology India Private Limited, Bangalore, Karnataka, India; 5 Indraprastha Institute of Information Technology Delhi, New Delhi, India; 6 Academy of Scientific and Innovative Research (AcSIR), Ghaziabad, Uttar Pradesh, India; Victoria University of Wellington, NEW ZEALAND

## Abstract

The variability of clinical course and prognosis of COVID-19 highlights the necessity of patient sub-group risk stratification based on clinical data. In this study, clinical data from a cohort of Indian COVID-19 hospitalized patients is used to develop risk stratification and mortality prediction models. We analyzed a set of 70 clinical parameters including physiological and hematological for developing machine learning models to identify biomarkers. We also compared the Indian and Wuhan cohort, and analyzed the role of steroids. A bootstrap averaged ensemble of Bayesian networks was also learned to construct an explainable model for discovering actionable influences on mortality and days to outcome. We discovered blood parameters, diabetes, co-morbidity and SpO2 levels as important risk stratification features, whereas mortality prediction is dependent only on blood parameters. XGboost and logistic regression model yielded the best performance on risk stratification and mortality prediction, respectively (AUC score 0.83, AUC score 0.92). Blood coagulation parameters (ferritin, D-Dimer and INR), immune and inflammation parameters IL6, LDH and Neutrophil (%) are common features for both risk and mortality prediction. Compared with Wuhan patients, Indian patients with extreme blood parameters indicated higher survival rate. Analyses of medications suggest that a higher proportion of survivors and mild patients who were administered steroids had extreme neutrophil and lymphocyte percentages. The ensemble averaged Bayesian network structure revealed serum ferritin to be the most important predictor for mortality and Vitamin D to influence severity independent of days to outcome. The findings are important for effective triage during strains on healthcare infrastructure.

## Introduction

The World Health Organization (WHO) declared the outbreak of coronavirus disease 2019 (COVID-19) as a global health emergency of international concern. Originating in Wuhan, China, the disease has spread to the rest of the world. As of 24^th^July, 2021, over 31 million confirmed cases of COVID-19 have been detected in India alone. Due to the sudden spike in the number of cases, healthcare systems across the world including India’s are under tremendous pressure for making tough decisions in resource allocation among affected patients. Early risk stratification through identification of key biomarkers is important as it holds potential for understanding the relative severity among infected patients sub-group and hence possible help in the decisions for better use of the healthcare infrastructure.

COVID-19 is a highly contagious respiratory infection with varying symptoms that include fever, dry cough, nasal congestion and breathing difficulties [1,2]. In more severe cases, it can cause pneumonia, severe acute respiratory syndrome, cardiac arrest, sepsis, kidney failure and death [3,4]. WHO classifies the risk into the following categories: critical, severe, and moderate/mild. By definition, critical patients require ventilation, severe patients require supplemental oxygen, moderate patients have pneumonia but do not require oxygen, and mild patients only have upper respiratory tract infection. The cause of death is generally respiratory failure, but few deaths have been caused by multiple organ failure (MOF) or chronic co-morbidities [2,5]. Those at a higher risk are the elderly and people with co-morbidities, such as cardiovascular diseases and diabetes [6,7]. However, symptoms at onset are relatively mild and a significant proportion of patients do not show apparent symptoms prior to the development of respiratory failure [2,5]. Clinically, this makes it difficult to predict the progression of severity in patients until respiratory failure develops. Early risk prediction and effective treatment can reduce mortality as well as help prioritize healthcare [8]. Artificial intelligence (AI) based solutions may help in clinical decision-making by providing predictions that are accurate, fast, and interpretable. Recent studies have used various machine learning algorithms for analyzing COVID-19 patients’ clinical data and providing disease prognosis [9–11]. Studies have also been conducted to compare the performance of different machine learning algorithms for multivariable mortality risk prediction [12–14]. Kuno et al. built a model based on Light Gradient Boosted Machine (LGBM) for predicting in-hospital mortality of COVID-19 patients administered with steroids and remdesivir. Hao et al. [15] examined COVID-19 patients admitted in Massachusetts to predict level-of-care requirements based on clinical and laboratory data. They compared machine learning algorithms (such as XGBoost, Random Forests, SVM, and Logistic Regression) and predicted the need for hospitalization, ICU care, and mechanical ventilation. The most effective features for hospitalization were vital signs, age, BMI, dyspnea, and comorbidities. Opacities on chest imaging, age, admission vital signs and symptoms, male gender, admission laboratory results, and diabetes were the most effective risk factors for ICU admission and mechanical ventilation. Xie et al. [16] used multivariable logistic regression for the classification task through identifying SpO2, lymphocyte count, age and lactate dehydrogenase (LDH) as the set of important features. A nomogram was created based on these features to deliver the probability of mortality. Ji et al. [17] built a scoring model, named as CALL, for prediction of progression risk in COVID-19 patients from Chinese hospitals. They used Multivariate Cox regression to identify risk factors associated with progression, which were then incorporated into a nomogram for establishing a prediction scoring model. Co-morbidity, older age, lower lymphocyte count, and higher lactate dehydrogenase were found to be independent high-risk factors for COVID-19 progression. Yan et al. proposed an interpretable mortality prediction model for COVID-19 patients [18]. They analyzed blood samples of 485 patients from Wuhan, China, and created a clinically operable single tree through XGBoost. The model used three crucial features lactate dehydrogenase (LDH), lymphocyte (%) and C-reactive protein (CRP). The decision rules with the three features and their thresholds were devised recursively. This provided an interpretable machine learning solution with at least 90% accuracy. Karthikeyan et al. [19] analyzed the same dataset through comparing various machine learning algorithms. XGBoost feature selection and neural network classification yielded the best performance with the important biomarkers selected as neutrophil (%), lymphocyte (%), LDH, CRP and age. However, detailed studies on risk stratification and mortality prediction using hospital admitted COVID-19 Indian patients’ clinical data needs a closer look. This becomes especially relevant as India was recently swamped with the second COVID-19 surge. At the same time, risk stratification based identification of biomarker/s can be prepared for preparedness for possible future waves.

Most machine learning based risk stratification and mortality prediction algorithms analysed patients from China or the USA. Studies have suggested that the virus has different variants of concern (VOC) around the globe due to mutations [20–23]. Moreover, the physiologic response to the virus and the eventual course of disease also depends on regional factors such as population characteristics and hospital treatment regimen. Hence, the studies are not universally applicable and it is critical to examine cohorts from India to aid the Indian healthcare systems. In this study, patients with confirmed COVID-19 infection from a hospital cohort in New Delhi, India were examined to identify the key features affecting severity and mortality. The machine learning models built using these key features can aid in risk stratification and mortality prediction. A comprehensive comparison between the cohorts from New Delhi and Wuhan [18] has also been done to understand the cohort-specific differences. Finally, models that can help discover actionable influences and potential causal mechanisms are important to discover actionable influences in complex decision making scenarios [24–26]. To this end, a directed acyclic graphical model (Bayesian network) approach was taken to infer and visualize the effect of the potential influencers for decision making in the New Delhi cohort.

## Methods

### Data acquisition and participants

The data in this study was collected from hospital admitted patients with confirmed diagnosis of COVID-19 at Max group of Hospitals in New Delhi, India between June 3rd and October 23rd, 2020. The patient records were collected and anonymized at the data warehouse of CSIR-IGIB. The use of collected data in this study has been approved by ethics committees of both Max Hospital and CSIR-IGIB. An informed and written consent was obtained from the participants themselves or from a legal guardian for participants under the age of 18. A total of 544 patients with a clear final outcome were considered in our study. Among these, diagnostic lab reports were available as a time series of test results. The data collected contains 357 distinct parameters (or biomarkers) that include vitals, symptoms, co-morbid conditions and lab reports from 161 different tests along with the medicines administered for treatment. Multiple tests were recorded for each patient during their stay at the hospital, varying from 1 to 134 records per patient. All methods and experiments were carried out in accordance with relevant guidelines and regulations.

### Risk stratification and statistical analysis

Patients were categorized into risk levels-based on the severity of their condition during their stay at the hospital. For patients with no clinical record referring to their severity level, their severity was inferred from the corresponding level of respiratory support required by the patient, as correlation between COVID-19 severity and hypoxemia being a well-documented phenomenon in multiple studies [27–29]. Considering the size of the dataset and the levels of respiratory support provided, all the patient were categorized into two levels, mild and severe, where all patients who died or who were under some form of respiratory support or whose condition was specifically mentioned to be severe were categorized into severe/high risk group and all the remaining patients were put under mild/low risk group. The resulting dataset follows the data distribution as shown in **Table 1**.

**Table 1:** Distribution of the number of patients across various classes.

Data Distribution			
Risk Category	Quaternary Stratification	Mortality	Binary Stratification
**Home Quarantined**	8 (1.47%)	483 (88.79%)	244 (44.85%)
**Hospitalized**	236 (43.38%)		
**On Respiratory Support**	239 (43.93%)		300 (55.15%)
**Died**	61 (11.21%)	61 (11.21%)	

The 15 most frequent tests corresponding to 38 biomarkers were selected for analysis based on the availability of clinical data. Five biomarkers—WBC count, neutrophil lymphocyte ratio (NLR), lymphocyte monocyte ration (LMR), neutrophil monocyte ratio (NMR), and platelet to lymphocyte ratio (PLR) were manually calculated from various blood cell counts available owing their reported importance in predicting mortality due to COVID-19 [30,31]. In our study, 209 unique co-morbid conditions were observed in patients. To aggregate the co-morbid conditions as per known effects of COVID-19on organ systems *vis-à-vis* respiratory [32], cardiac [33], nervous [34], renal [35], and hepatic [36], we grouped all the co-morbid conditions into 11 groups based on systemic and multi-systemic diseases [7]. This also prevents increasing the chances of over fitting due to increase in dimensionality. The groups being respiratory, nervous, circulatory, renal, thyroid, liver related and cancer, hypertension, diabetes, hyperlipidemia and others as shown in S1 Table. This information was encoded into 11 binary features, each representing one group where a sample assumes a value one if the patient has one or more co-morbid conditions that fall into that group. To incorporate and analyze the effects of medical prescriptions, the information regarding prescription of steroids and antiviral drugs was encoded into two binary features.

This leads to 70 unique parameters measured which include 11 grouped co-morbid conditions, 14 clinical parameters, 2 RT-PCR parameters and 43 lab test results. An exhaustive list of categorical parameters can be found in S1 Table and continuous parameters can be found in S2 Table. To evaluate the significance of each parameter considered for risk stratification and mortality prediction, we calculated the p-value using the Chi-Squared test [37] for the categorical features and using the ANOVA f-value test for the continuous features.

### Comparison with Wuhan cohort based ML model

To understand the accuracy difference in mortality prediction of machine learning models across different populations, we evaluated how machine learning models trained on non-Indian cohorts perform in predicting mortality on the Indian cohort. We used the best performing model reported by Karthikeyan et.al [19] for predicting mortality using data from Wuhan, China [18] to examine its applicability on the Indian cohort. The Wuhan cohort comprises of data collected from 375 patients who were admitted to Tongji Hospital, Wuhan. The model evaluated is a neural network trained to predict mortality from CRP, LDH, neutrophil (%) lymphocyte (%) and age. For predicting mortality in Indian Cohort using the same model, we selected 3092 data points where at least 3 of the required 5 features were present. KNN imputation was done to take care of the missing features.

To understand the plausible cause of difference in prediction accuracy of machine learning models across populations, we explored the differences between Wuhan and New Delhi cohorts in key biomarkers across survivors and the dead [18,19,38]. We choose mortality as the indicator for comparison as it does not depend on subjective labeling. The feature density histograms were analyzed to examine the variations in biological parameters across survivors and the dead between cohorts of Wuhan and New Delhi. The Kolmogorov-Smirnov test (K-S test) [39] was used to analyze variations in the density distributions of the important biomarkers between both classes across cohorts. The K-S test is a non-parametric test that quantifies the distance between the empirical distributions of samples sampled from two distributions.

### Machine learning pipeline

**Fig 1:**
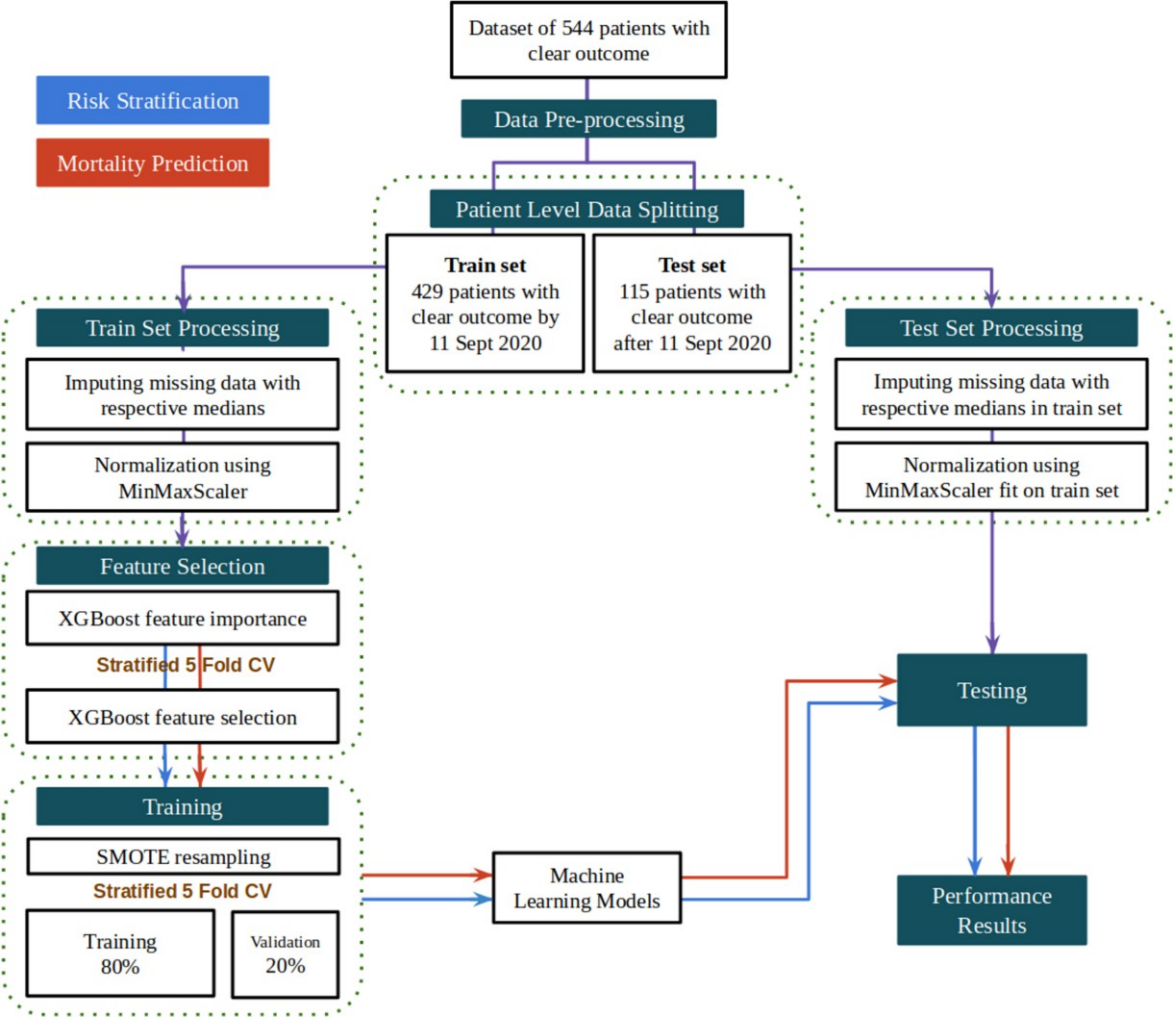
Machine learning pipeline for the development of the risk stratification and mortality prediction.

Overall pipeline used in this study for risk stratification and mortality prediction is depicted in **Fig 1.** We compared several machine learning algorithms namely XGBoost, random forests, Support Vector Machine (SVM) and logistic regression for evaluating their predictive performance. A detailed account of the step-by-step procedure is presented in the following sections.

### Data pre-processing

For each patient in the dataset, there were multiple lab test results recorded on different days before the final disease outcome. We have considered each individual recorded test result as a unique data point for training and testing as has been done before [18,19]. Each sample has a dimensionality equal to the number of unique parameters measured across all lab tests considered for the analysis. The values in a sample are filled in with the test results that a particular sample represents and the rest of the values are left empty. These parameter values that are left empty are imputed with the nearest value of the parameter from the patient’s past test results. Some samples may still have missing parameters if a patient does not undergo a particular test. Such missing values are imputed with the median of the respective parameter across the train set. Patient demographics and vitals data were recorded once per patient and were added to each sample where they are kept the same for all the samples of a particular patient. This leads to 15648 samples from 544 patients where each sample contains 70 unique parameters.

To build and validate machine learning models we split patient sub-groups with respect to the day of outcome. 429 patients with clear outcome by 11 September 2020 were considered for model development, and the remaining 115 patients were considered as a part of a holdout test set. This method of splitting is adopted as models developed will be used to aid future patients where it is known that the COVID-19 and responses of its infected patients may change with time [20–23]. The day wise distribution of samples in both the train and test sets for risk stratification and mortality prediction is shown in S1 and S2 Figs, respectively.

### Feature selection

Among the 70 features chosen for analysis, selecting the most influential biomarkers for risk stratification and mortality prediction by eliminating redundant or unimportant parameters is crucial to avoid over-fitting when the size of the dataset is small. Moreover, a lower number of features would mean economical and faster tests for efficient risk profiling given the high influx of patients on a daily basis and subsequently increased efficiency of the decision-making process of the healthcare systems. The relative importance of a biological parameter provided by an XGBoost classifier fit on the training data for a particular task is used as the measure of importance for selecting features. XGBoost is a powerful decision-tree-based ensemble algorithm that uses a gradient boosting framework and estimates features that are the most discriminative of model outcomes [40]. The relative importance of each feature is determined by its accumulated use in each decision step in each tree of the ensemble.

The number of features to utilize for model training was obtained by iteratively training an XGboost model on a collection of the top K most important features while increasing K by 1 during each iteration. The collection of features that achieved the best performance for 5-fold cross validation on the training set was considered as the set of key features to train the final models. The feature importance was obtained separately for the binary risk stratification and mortality prediction models. The classification performance for selecting the optimal set of features is evaluated using AUC score for risk stratification and average precision score for mortality prediction. Average precision score is used for mortality prediction due to the imbalance of samples representing fatal cases in mortality prediction.

### Training

After obtaining the collection of important features, duplicates that arose due to the elimination of less important features were removed from the train set. The set was then normalized to a range of 0–1 using min-max scaler to avoid any biases due to differences in scales across parameters. The train set was then resampled using the SMOTE algorithm to reduce bias that may arise due to the class imbalance observed. The SMOTE algorithm was chosen to generate synthetic samples of the minority class due to its good performance. Various algorithms were trained and compared on the resampled dataset to classify the samples depending on the task, either risk stratification or mortality prediction, with their respective feature set. We also built another set of models trained on only patient vitals to gauge the prediction performance that can be achieved with data acquired before blood test results.

### Testing

The hold out test data of 115 patients was normalized with min-max scaler to a range of 0–1 using the min-max statistics obtained from the training set. Then the models built were evaluated on the test set. We report the AUC and F1-scores of the algorithms as the mean and standard deviation of performance of trained models from 5-fold cross validation on the test set. The model achieving the best performance was then tested and analyzed on the set of samples corresponding to each individual day for a period of 14 days before the final outcome to observe relevant trends.

### Evaluation metrics

The following metrics were recorded to assess the predictive performance of the supervised models. Formulae for the calculation of all metrics are given below. Here, TP, TN, FP, and FN stand for true positive, true negative, false positive and false negative rates, respectively.

#### AUC (Area under ROC curve)

AUC measures the area under the receiving operator characteristic (ROC) curve, which plots true positive rate against false positive rate. AUC is also commonly used in situations where the data has imbalanced classes, as the ROC measures performance over many different thresholds.

- True Positive Rate (TPR): This measures how often the model predicts that a patient will survive when the person survives.

$$TPR=\frac{TP}{TP+FN}$$

- False Positive Rate (FPR): This measures how often the model predicts that a patient survives when the person actually does not survive:

$$FPR=\frac{FP}{FP+TN}$$

#### F1 score

The F1 score measures the harmonic mean of precision of recall and is often preferred to accuracy when the data has imbalanced classes:

$$F1\;Score=\frac{2*Precision*Recall}{Precision+Recall}$$

where,

$$Precision=\frac{TP}{TP+FP}$$

and,

$$Recall=\frac{TP}{TP+FN}$$

### Structure learning

A data-driven structure learning approach was taken to learn actionable interventions for clinical decisions. Eleven Bayesian networks were ensembled to create the consensus graph. Each network was learned from a bootstrapped sample of the data, hence expected to be slightly different for each run. Hill climbing optimization algorithm was used to learn each network using the Akaike Information Criterion score. Majority voting was used to construct the consensus Bayesian network with the condition that edges with consistent presence and direction in at least 6 out of 11 networks were selected. The consensus network was then parameterized with conditional probabilities using junction tree algorithm, thus marginal probabilities and conditional probabilities were inferred using Exact Inference method of Bayesian network inference [41]. Structure learning, inference, and visualization were carried out using *wiser* [25] package in R [42].

## Results

### Patients’ clinical diversity across disease sub-phenotype

**Fig 2:**
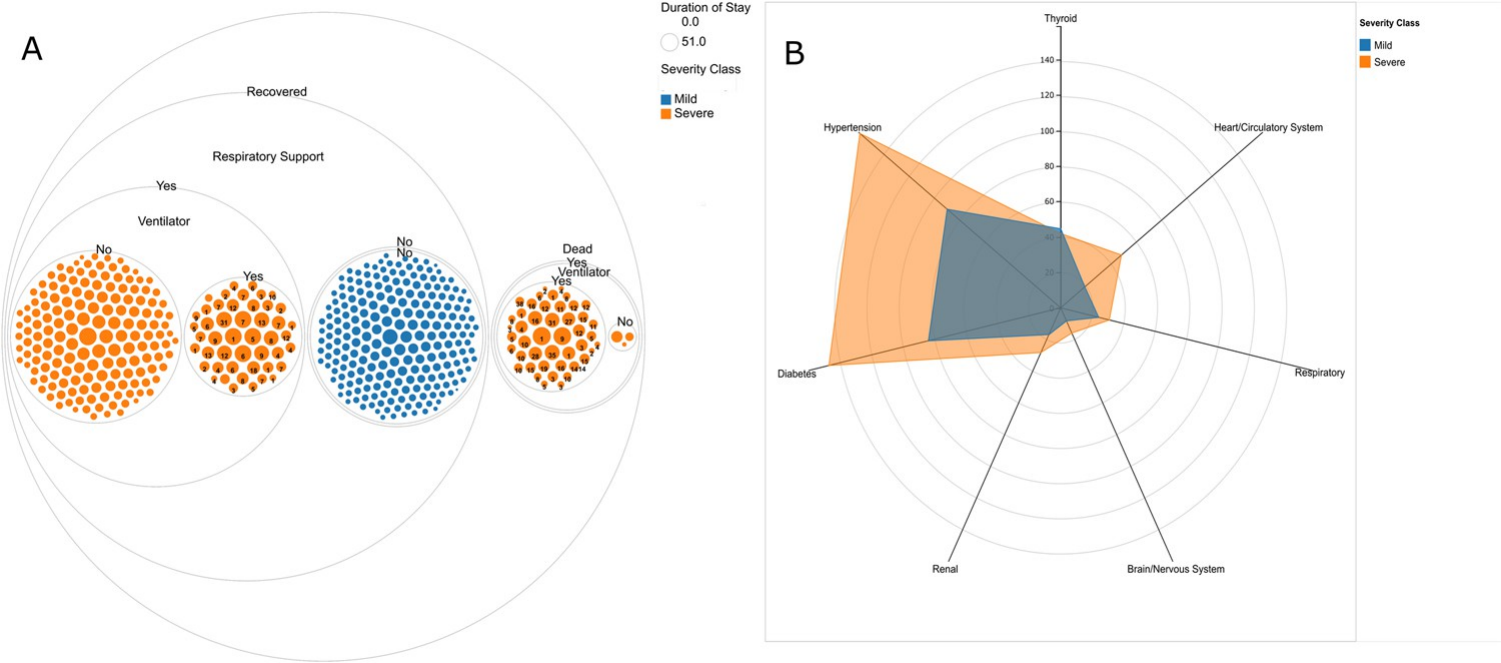
Clinical sub-phenotypes and the co-morbidity feature diversity. **(A) Clinical sub-phenotype diversity of the COVID-19 patients.** The patients are grouped into Recovered and Dead. Each circle represents individual patients; the color of the circle indicates the severity of the patients whereas the size of the circle represents duration of hospital stay. The numbers on the circle represents the duration in ICU. **(B**) **Presence of different co-morbid conditions in mild and severe patients.** It represents a comparative view of the co-morbidities, patients with mild severity are represented by blue color, while ones with severe COVID-19 infection are represented by orange.

Comparative analysis of clinical features between low and high-risk patients was carried out. S1–S4 Tables show the diversity in categorical and continuous features between high and low risk groups as well as between survivors and the dead. **Fig 2A** captures the distribution of patients across respiratory/ventilator support and the hospital stay. The KS test showed that none of the continuous features followed a normal distribution and hence the medians and interquartile ranges are reported. The patients’ age ranged between 9 and 98 years with the median age of 58 (48–66) years. The median age for the high-risk patients was 61 (53–68) years while for the low-risk patients it was 53 (41–64) years. Out of the 544 patients, 164 (30.15%) were females while 380 (69.85%) were males. The blood clotting (D-dimer & ferritin), inflammation (CRP, LDH) and immune features (NLR, LMR, NMR, PLR and IL6) were significantly different for the low and high-risk groups. However, a significant overlap was observed in most of the parameters, both when comparing the high-risk vs. low-risk and survived vs. dead categories precluding the possibility of the development of simple classification models. We also observed differential abundance of co-morbidities across mild and severe patients (**Fig 2B**). The increased incidence of hypertension, diabetes and cardiovascular co-morbidities was seen in severe COVID-19 patients in our cohort.

### Performance of Wuhan cohort trained model on the Indian cohort

**Fig 3:**
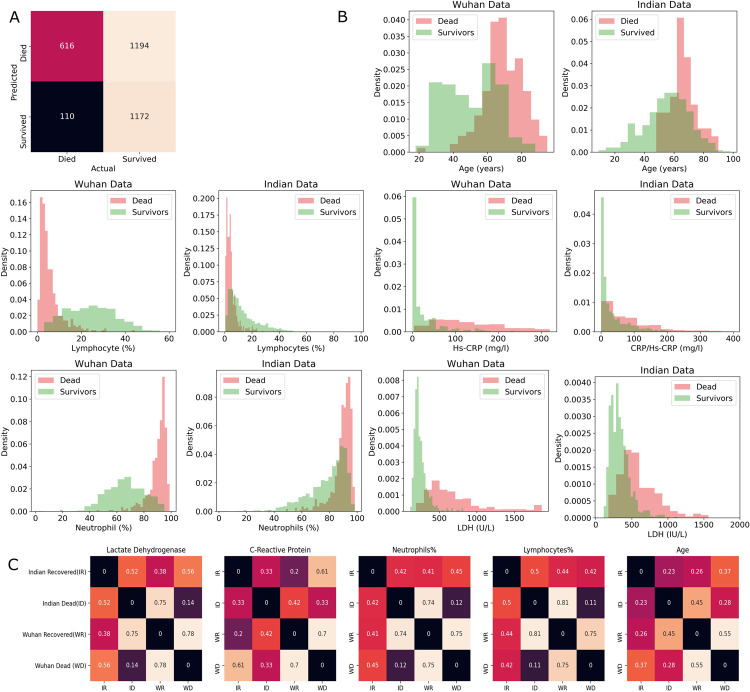
**(A) Confusion matrix of neural net trained on Wuhan data and tested on Indian data.** This was done by comparing actual and predicted mortality of patients in the dataset. **(B) Comparison of the normalized histogram plots of important features useful for predicting mortality from Wuhan and Indian Cohorts.** It shows the comparative distribution of clinical parameters between death and survival cases. **(C) Pair-wise distances between distributions of important features across the Indian vs. Wuhan survived and dead classes.** Distance values were calculated through Kolmogorov–Smirnov test.

Karthikeyan et.al. [19] built a neural network that predicted mortality in Wuhan cohort with an accuracy of 96.5%, using only five parameters, age, lymphocyte (%), neutrophil (%), LDH and CRP. The same model when tested on the New Delhi cohort (current dataset) predicted mortality with an accuracy of only 58%. The drop in performance of the model when tested on the Indian cohort shows that there is a significant difference between the two cohorts. **Fig 3A** demonstrates that the Neural Net was performing much better in identifying the patients who died (precision 84.85%) over those who survived (precision 49.54%). This suggests that the patients who were expected to die based on the findings from Wuhan data were actually surviving in the Indian cohort.

To understand the difference between cohorts, we compared the feature density histograms of Indian and Wuhan cohorts **(Fig 3B)**. It was observed that survival of patients with LDH in the range 500-1000units per liter (U/L) is much higher in Indians compared to Wuhan. It can also be observed that there are almost no survivors with an LDH value greater than 800U/L in the Wuhan cohort while patients with LDH values of even about 1000U/L have survived in the Indian Cohort. The survivability of patients with CRP greater than 50U/L is higher in the Indian cohort compared to Wuhan. Similar conclusions can be drawn with Indian patients having relatively lower lymphocyte (%) and higher neutrophil (%). This is interesting as the likelihood of survival with higher neutrophil (%) or lower lymphocyte (%) is much lower [43].

Various matrices with two sample K-S statistics that measure pair-wise distances between distributions of important biomarkers of survivors and the dead across Indian and Wuhan cohorts has been shown in **Fig 3C**. It is observed that the distance between distributions of the Indian Recovered (IR) and Indian Dead (ID) is significantly lower compared to the distance between the distributions of the Wuhan Recovered (WR) and Wuhan Dead (WD) for all the five biomarkers. This is mainly due to the differences between distributions of recovered across Indian and Wuhan as the distance between the cohorts of the dead is low and the distance between cohorts of the recovered is high. This suggests that Indian patients who were at risk of death (with extreme neutrophil and lymphocyte percentages) have survived.

### Characteristics of risk stratification models

XGboost was used to rank features based on the contribution of each feature to the performance in risk stratification. S3 Fig shows the list of the top 25 features sorted in descending order with respect to their relative importance in risk stratification. The 11 features that were selected to train the models in the order of their importance are absolute neutrophil count, LDH, lymphocyte (%), neutrophil (%), diabetes, ferritin, INR, interleukin-6 (IL-6), SpO2, absolute eosinophil count and packed cell volume. S4 Fig shows the density distributions for the top 4 features identified.

**Fig 4:**
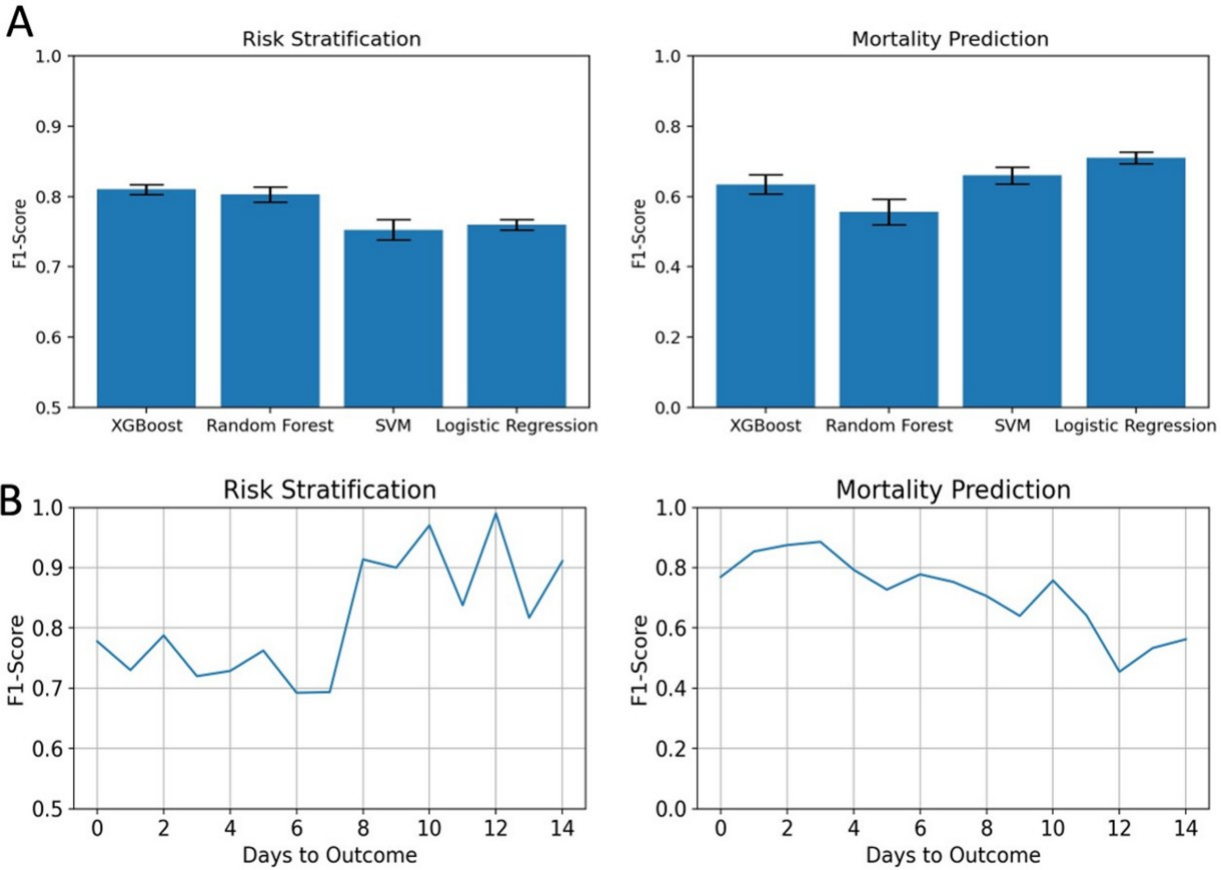
(A) Comparison of F1 scores for various machine learning models that use patient vitals and lab test results. (B) Performance of the ML models with respect to number of days to outcome.

Comparison of the performance of various algorithms showed XGboost algorithm to perform the best with an F1-score of 0.810±0.01 as seen in **Fig 4A**. The model also yielded better AUC (0.833±0.01) and average precision (0.891±0.01) (S5 Table). The confusion matrix of predictions from an XGboost model trained on the entire train set is shown in S5 Fig. We also evaluated how the performance of model changes with days to outcome, where the day of outcome is either the day of discharge from the hospital or the day of death. **Fig 4B** shows that the performance of the risk stratification model decreases as the samples approach the day of outcome. This suggests that the feature difference between low risk and some high-risk patients who are recovering is decreasing towards the day of outcome. However, the performance of the mortality prediction model increases towards the day of outcome. Hence, selective use of these two models depending on the number of days from infection may be effective.

Further, we trained and evaluated models with only patient vitals, co-morbidities, and medication information to evaluate the predictive performance that can be achieved without lab test results. S6 Fig shows the F1 scores of various models that were built to use only these patient’s information. The random forests algorithm performed the best with an F1 score of 0.76±0.02. The important features selected were administration of steroids, SpO2, diabetes, thyroid problems, presence of any other co-morbidities, weight, temperature, respiration rate, hypertension, and BMI.

### Characteristics of mortality prediction models

Similar to risk stratification, the features for mortality prediction were also analyzed for. We observed the top 25 features with respect to their relative importance in mortality prediction, sorted in descending order (S7 Fig). The nine features that were selected to obtain the results in the order of their relative importance are D-dimer, ferritin, lymphocyte (%), NLR, WBC, Trop I, INR, IL-6 and LDH. A representative density distribution for the top 4 identified features has been shown in S8 Fig.

Among the models tested, Logistic regression performed the best with an F1-score of 0.710 0.02 (**Fig 4A**). The model also yielded better AUC (0.927 0.01) and average precision (0.801 0.02) (S6 Table). We also observed that the performance of the model increases as the samples approach the day of outcome as (**Fig** 4B). We trained and evaluated models with only patient vitals, co-morbidities, and medication information to evaluate the predictive performance that can be achieved with data excluding lab test results. S6 Fig shows the F1-scores of various models that were built using the selected patient information. SVM performed the best with an F1 score of only 0.34 0.03. The important features selected were hypertension, co-morbidities related to liver, cancer, SpO2, administration of antivirals and respiration rate.

### Possible role of medication (steroids)

**Fig 5:**
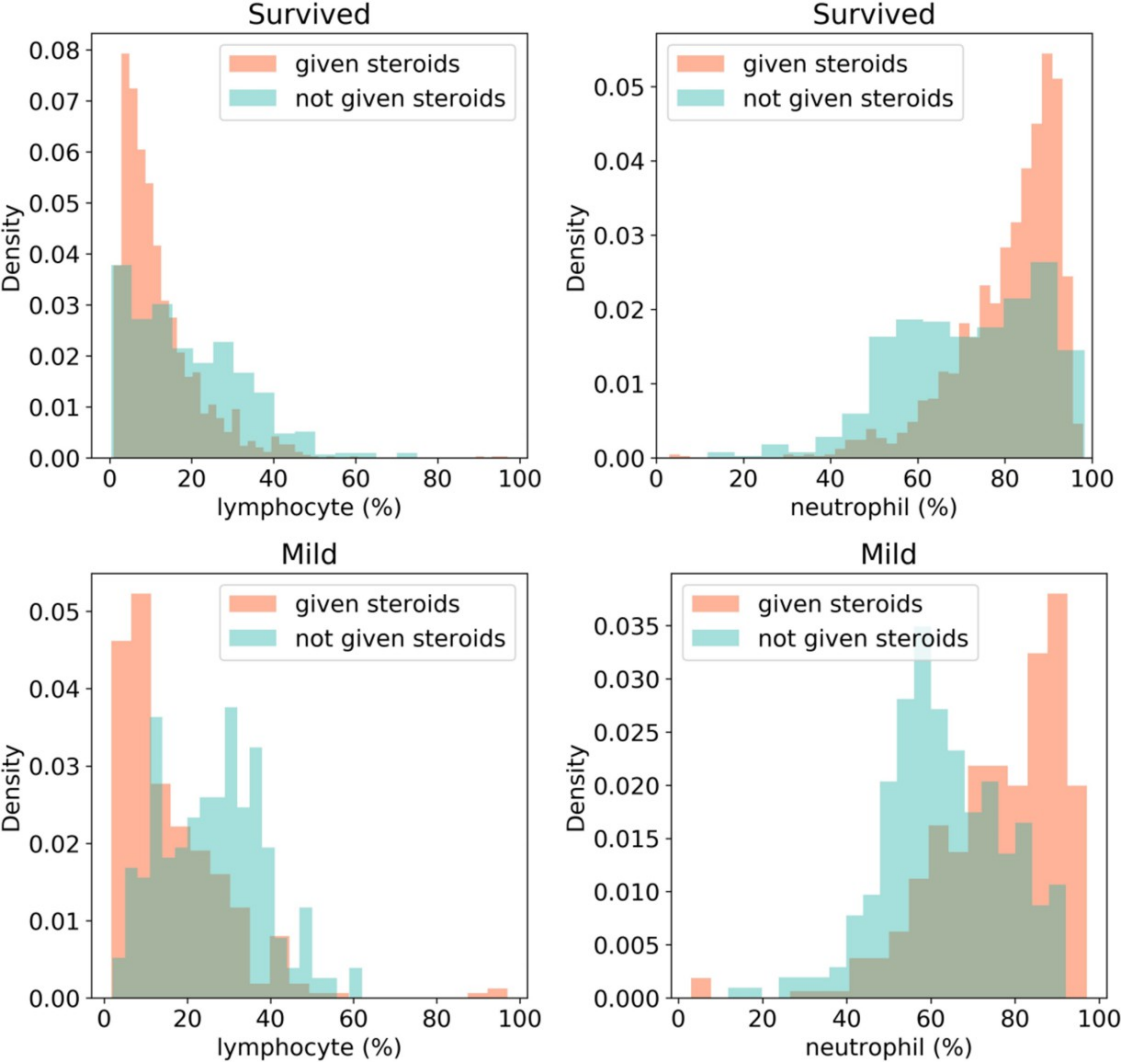
Distribution plots for lymphocyte (%) and neutrophil (%) in steroid administered and non-administered patients having mild and severe disease.

Closer look at the medication revealed that steroids have used in majority of the patients. Whether that has potential role in disease outcome? We compared the differences in neutrophil and lymphocyte percentages across patients who were administered steroids and patients who were not. Of the 544 patients, 338 (62.13%) patients were administered steroids. It was observed that Methylprednisolone was the most widely administered steroid that was given to 262 different patients, followed by Dexamethasone (89 patients), Prednisolone (11 patients) and Hydrocortisone and Triamcinolone were given to one patient each. It is to be noted that there were instances where a single patient was administered with more than one of these. Fig 5 shows the density histograms of neutrophil and lymphocyte percentages for survivors and mild patients. It is observed that a higher proportion of the survivors and mild patients who were administered steroids had extreme neutrophil and lymphocyte percentages indicating that administration of steroids may have had an impact on the patient outcome.

### Explainable AI framework for guiding actions

**Fig 6:**
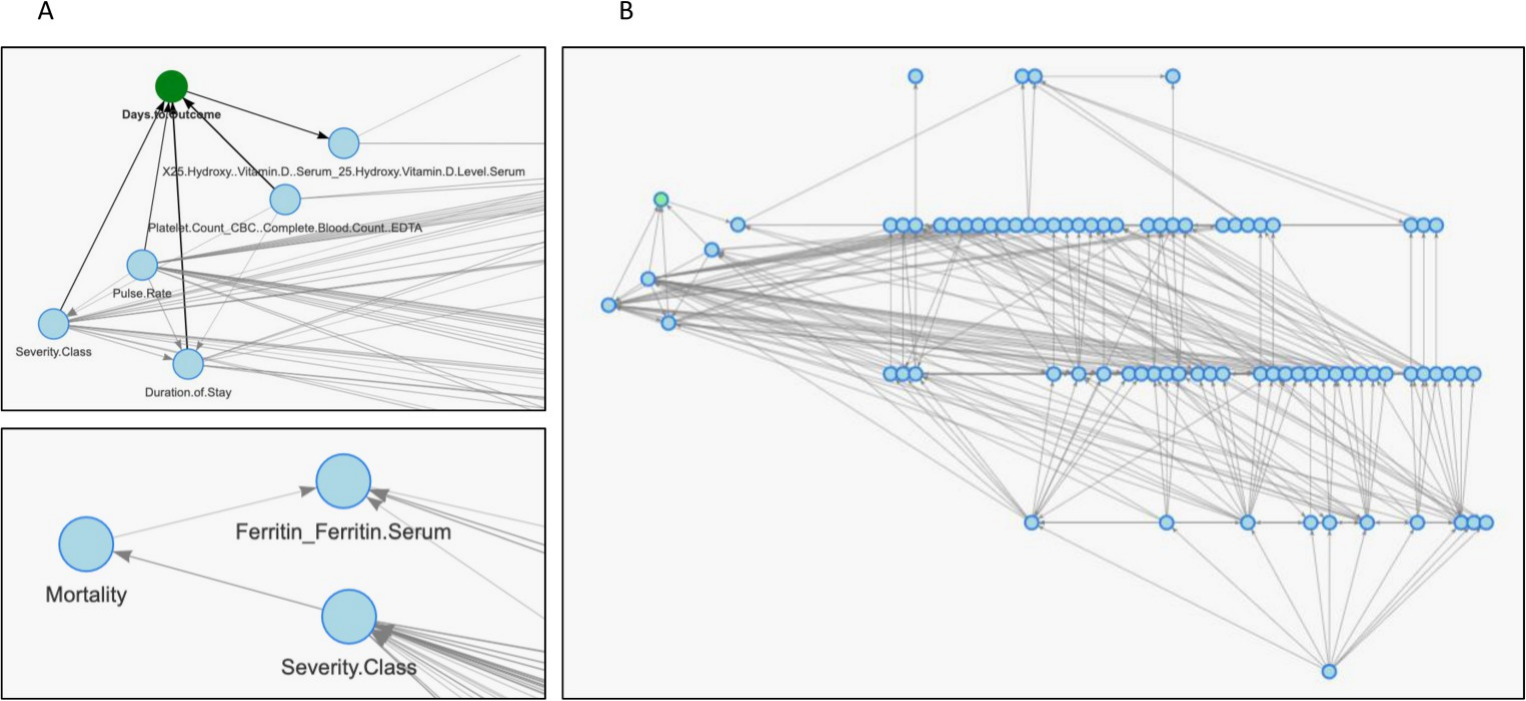
Explainable AI model to discover and quantify actionable factors. A zoomed in portion (A) of the complete structure, (B) learned as directed acyclic graph revealed the key factors for *Mortality* and *Days to outcome*. Each node is a variable, and the edges represent direction of probabilistic influence learned from data. In the Indian dataset, model inference revealed that *Serum Ferritin* was the most important predictor of *Mortality*. Further, high levels of 25-hydroxy vitamin D delayed the *Days to outcome* independent of *Severity Class*, thus indicating a potential protective effect despite the outcome being primarily determined by severity. The explainable framework is proposed to be used for reasoning and decision-making in the Indian settings. Here we take two examples of outcomes of interest, i.e. mortality and days to mortality. The change in percentage probability of the outcome in a certain interval (e.g. high mortality or lower number of days to death) was inferred conditioned upon the learned associations in the network. S7 Table shows the inferences using the Exact Inference algorithm on the learned structure, which quantify the key influences.

We learned the structure of model in an explainable AI approach to serve as a framework for decision-making in the Indian dataset. This requires the models to discover confounding, mediation and competing influences. These influences are discovered and transparently revealed as network motifs, i.e., fork, chain and collider network edges in the graphical model [41]. The overall mortality was primarily indicated by severity of illness and ferritin levels in the blood. Ferritin was found to be the single most important predictor of mortality with a 75% increase in probabilistic influence for death when high levels of ferritin were present. Among many novel influences discovered, our explainable AI model revealed disease severity, platelet count, pulse rate and serum Vitamin D levels. Importantly, the latter was independent of disease severity. Setting the Vitamin D level as high in the model led to a 19% increase in probability of increase in days to outcome (mortality). The overall mortality was primarily indicated by severity of illness and ferritin levels in the blood. Our model not only confirms these findings but also quantifies these in a contextual network structure that can be deployed as a model for New Delhi settings **(Fig 6)**.

## Discussion

**Fig 7:**
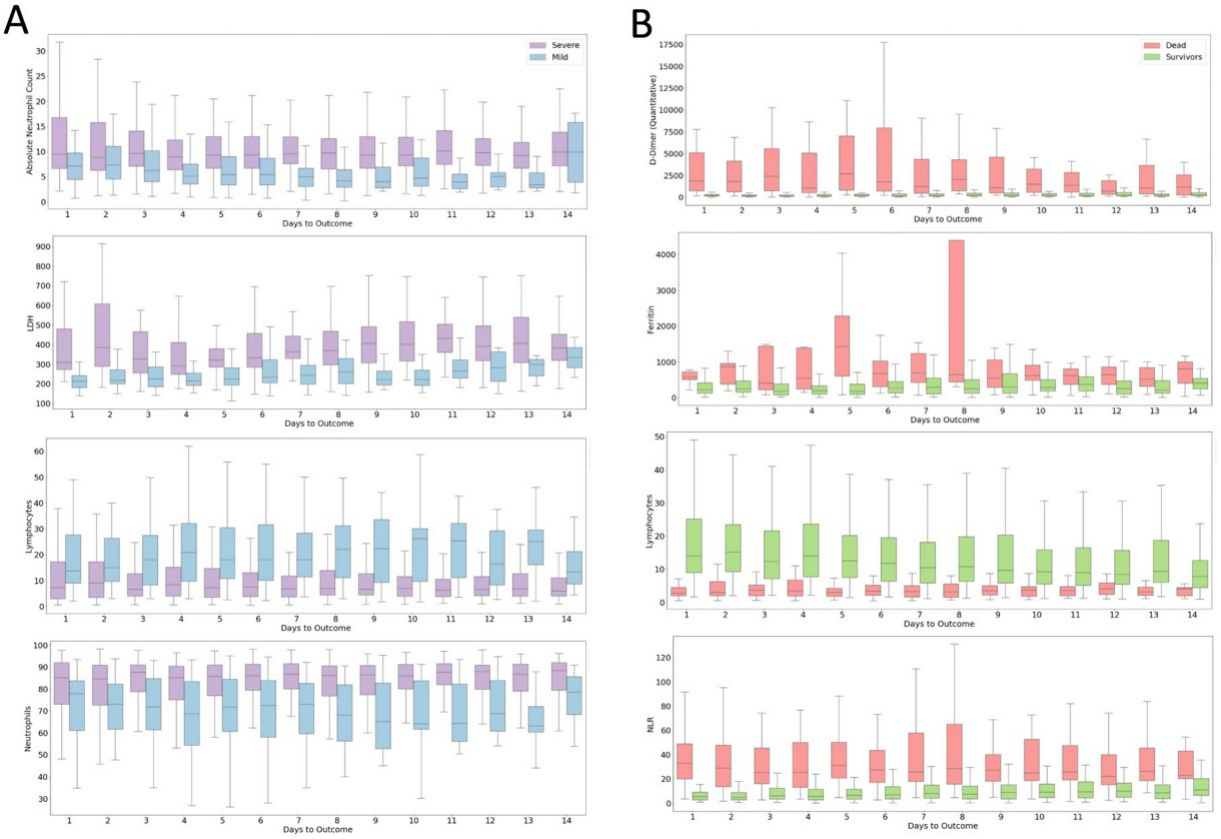
Biomarker variations in different patient classes in due course of disease progression by (A) risk (B) mortality parameters. Showing consistent separation of biomarker levels in mortality prediction parameters and a decrease in separation of risk prediction parameters.

COVID-19 has spread around the globe and the need for fast and effective resource allocation is urgent, but very few studies have examined Indian cohorts. In this study, we analyzed 15648 samples of 544 patients, with confirmed diagnosis of COVID-19. Each sample contains 70 unique parameters including the grouped co-morbid conditions, patient vitals, patient demographic information, and lab test results. We found that existing mortality prediction models trained on Wuhan cohort cannot be directly used for mortality prediction on the Indian cohort due to cohort specific differences in response to COVID-19. We observed greater overlap between dead and survivors’ parameter/biomarker distributions in the Indian cohort than in Wuhan. It was observed that KS distance between distributions of WR and IR for neutrophil and lymphocyte percentages is comparatively high while the distance between the distributions of the dead (WD, ID) across the cohorts was low. This shows that the increased overlap in the distributions in the Indian cohort is primarily due to survivors. Patients in India recovered even when their neutrophil and lymphocyte percentages reached levels similar to the levels of patients who died in Wuhan. A probable reason for the low mortality in the Indian cohort may be the inclusion of steroids and immunosuppressant drugs in the treatment protocols early on in the timeline of the pandemic. Studies have shown that use of steroids like Dexamethasone lowered COVID-19 fatalities when administered to patients who require supplemental oxygen [44–47]. We observed a relation between the usage of these drugs and the survival of patients with extreme lymphocytes and neutrophils counts, which are associated with mortality (**Fig 7**) [18,19,38,48,49].

Machine learning models for risk stratification and mortality prediction were developed based on features extracted from Indian cohort. The important features for risk stratification included blood parameters, diabetes, comorbid condition and oxygen saturation level. On the other hand, mortality prediction is dependent only on blood parameters, inclusive of NLR, WBC and Trop I. Blood coagulation parameters (ferritin, D-Dimer and INR), immune and inflammation parameters (IL6, LDH and Neutrophil (%)) are common features for both risk and mortality prediction. Some of these features have been identified as predictors of the progression of the COVID-19 disease [18,19,38,49,50].

The best performing model for risk stratification on the Indian dataset was the XGboost classifier, which achieved an F1-score of 0.81±0.01 while Logistic regression yielded the best performance for mortality prediction with an F1-score of 0.71±0.02. We also examined the performance of these algorithms when trained on a dataset comprising of only vitals and clinical attributes, as these are features that can be acquired quickly and may aid in the initial decision-making process. The best performing models gave an F1 score of 0.76±0.02 for risk stratification and 0.34±0.3 for mortality prediction. The low performance of these models shows the importance of blood parameters in describing the progression of COVID-19.

We observed that the progression of COVID-19 is accompanied by hemocytometric changes with respect to the numbers of days to outcome (**Fig 7**). The final day of outcome was considered as it is a more stable reference point compared to the day of admission as a patient may be identified and admitted late in the progression of the disease. The patients who died showed elevated levels of D-dimer, ferritin and NLR, while lymphocyte (%) levels dropped. The separation of the biomarkers’ values between the two classes is observed to be consistent through the course of the disease. This shows their plausible significance in making predictions. Interestingly, the mortality prediction model performed better when nearing the day of outcome whereas the performance of the risk stratification model decreased as we move towards the day of the outcome. The differences between the survivors and the dead increase as the time progresses as survivors recover from the conditions whereas patients who die do not, making it easier for any predictive model to classify. The performance of risk stratification decreases as we move towards the day outcome because as patients recover the differences between low risk and high-risk candidates converge, making it more difficult for the model to classify.

Our study provides a preliminary assessment of the clinical course and outcome of Delhi patients. We intend to test these models in the future on larger data collected from multi-hospitals located in different geographic locations in India. As more data becomes available, the whole procedure can easily be repeated to obtain better models and more insights. Although we had a pool of about 70 clinical measurements, here our modelling principle is a trade-off between the minimal number of features and the capacity for good prediction, therefore avoiding overfitting. Nevertheless, studies done on other cohorts have also identified these features as key predictors [49].

**Fig 8:**
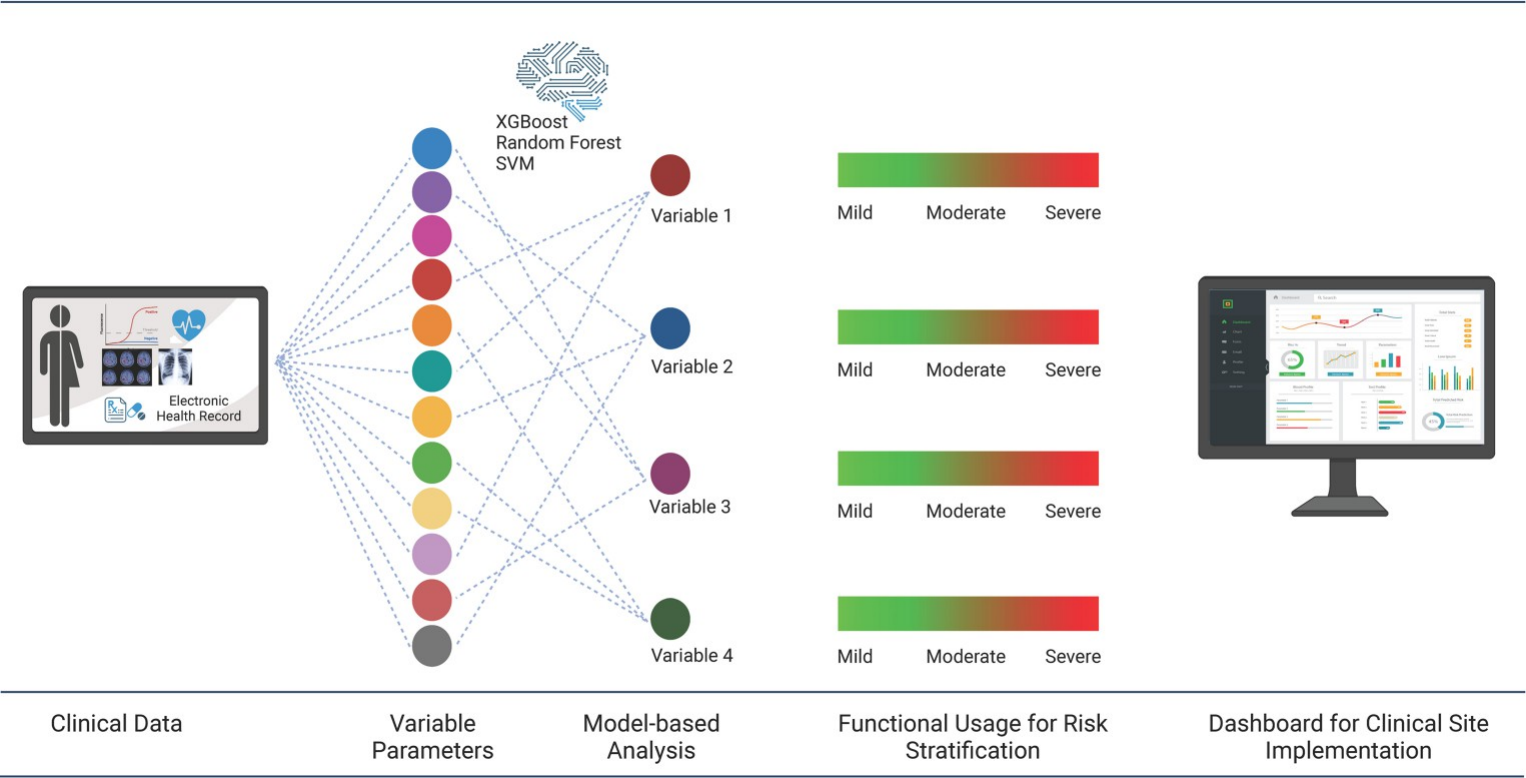
Model-based analysis of clinical data for risk stratification with potential clinical implementation. Machine learning model-based analysis of clinical data variables to identify parameters for risk stratification, and development of dashboard for implementation of the model at the clinical site.

The adoption of AI in healthcare is contingent upon building trust with the clinicians. Hence, models that can encode the complexity of interactions between predictors yet are transparent are crucial. In a recent systematic review of more than 400 AI models proposed for COVID-19 diagnosis from radiographs and scans, none were found to be reproducible and transparent enough to be deployed in clinical settings [51]. Hence, we constructed a framework for deployable, transparent and explainable model using the Bayesian graphical model approach. This approach reveals the proximate factors that have the most probabilistic influence on the outcome of interest and differs from the traditional feature selection approach by identifying the network motifs that encode confounding, mediation and competing effects. Using this approach, we discovered Ferritin to be an independent and single most important predictor of mortality, other than clinical severity. Other studies [52] have shown this before in other cohorts in United Kingdom [51] and our approach validated this finding in a completely different cohort using a smaller dataset through the use of explainable AI. We believe that such data-driven Bayesian networks, by the virtue of yielding pathway structures, can be contextualized to different settings using the Bayesian prior approach to structure learning. Therefore, our study provides an opportunity to converge the plethora of diagnostic variables observed in the early phases of disease into a few consequential parameters. These parameters can be assessed thereafter by the clinicians using a clinically operable standalone dashboard to effectively stratify patients. A brief outline of this approach and perspective is summarized in the illustration **Fig 8**.

## Conclusion

Accurate risk stratification and mortality prediction models based on vitals, co-morbidities and blood parameters will help in rapid screening of infected patients and hence in optimal use of the healthcare infrastructure. It is likely that cohort-specific difference may emerge due to the difference in demographic conditions and healthcare setting. This necessitates the development of population specific solutions. There is also a need to study the effectiveness of certain treatment protocols affecting mortality. Our study presents the first data collection effort to develop predictive models and to study feature differences and the possible effect of steroids in the Indian COVID-19 outcome. Risk stratification and mortality prediction models yielded good performance and AUC scores of 0.83 and 0.92 respectively. Hematological parameters are important features for risk stratification and mortality prediction models. The analysis showed that steroids might have played a role in patient survival with extreme neutrophils or lymphocytes. This study would help accelerate the decision-making process in healthcare systems for focused and efficient medical treatments.

## Supporting information

S1 Fig Sample distribution for risk stratification with time to outcome. (PDF)

S2 Fig Sample distribution for mortality prediction with time to outcome. (PDF)

S3 Fig Top 25 important parameters for binary risk stratification. (PDF)

S4 Fig Distribution plots for four most important features used for risk stratification. (PDF)

S5 Fig Confusion matrices for mortality prediction with logistic regression and Risk Stratification with XGBoost Classifier trained on entire train set. (PDF)

S6 Fig Comparison of F1 scores for various machine learning models that use only patient vitals. (PDF)

S7 Fig Top 25 important parameters for mortality prediction. (PDF)

S8 Fig Distribution plots for four most important features used for predicting mortality. (PDF)

S1 Table Categorical features for risk stratification. Medians and P-values are given for individual features.
(PDF)

S2 Table Continuous features for risk stratification. Medians and P-values are given for individual features.
(PDF)

S3 Table Categorical features for mortality prediction. Medians and P-values are given for individual features.
(PDF)

S4 Table Continuous features for mortality prediction. Medians and P-values are given for individual features.
(PDF)

S5 Table Performance of the developed machine learning algorithms in risk stratification reported as mean ± standard deviation. (PDF)

S6 Table Performance of the developed machine learning algorithms in mortality prediction reported as mean ± standard deviation. (PDF)

S7 Table The inferences using the exact inference algorithm on the learned structure. (PDF)

S1 Data (XLSX)

## References

[1] Zu ZY, Jiang MD, Xu PP, Chen W, Ni QQ, Lu GM, et al. Coronavirus Disease 2019 (COVID-19): A Perspective from China. Radiology. 2020 Aug;296(2):E15–E25. doi: 10.1148/radiol.2020200490 32083985 PMC7233368

[2] Huang C, Wang Y, Li X, Ren L, Zhao J, Hu Y, et al. Clinical features of patients infected with 2019 novel coronavirus in Wuhan, China. Lancet. 2020 Feb 15;395(10223):497–506. doi: 10.1016/S0140-6736(20)30183-5 31986264 PMC7159299

[3] Sanche S, Lin YT, Xu C, Romero-Severson E, Hengartner N, Ke R. High contagiousness and rapid spread of severe acute respiratory syndrome coronavirus 2. Emerging Infect Dis. 2020 Jul;26(7):1470–1477. doi: 10.3201/eid2607.200282 32255761 PMC7323562

[4] Ponsford MJ, Gkatzionis A, Walker VM, Grant AJ, Wootton RE, Moore LSP, et al. Cardiometabolic Traits, Sepsis, and Severe COVID-19: A Mendelian Randomization Investigation. Circulation. 2020 Nov 3;142(18):1791–1793. doi: 10.1161/CIRCULATIONAHA.120.050753 32966752 PMC7594537

[5] Wang D, Hu B, Hu C, Zhu F, Liu X, Zhang J, et al. Clinical Characteristics of 138 Hospitalized Patients With 2019 Novel Coronavirus-Infected Pneumonia in Wuhan, China. JAMA. 2020 Mar 17;323(11):1061–1069. doi: 10.1001/jama.2020.1585 32031570 PMC7042881

[6] Liu Y, Mao B, Liang S, Yang J-W, Lu H-W, Chai Y-H, et al. Association between age and clinical characteristics and outcomes of COVID-19. Eur Respir J. 2020 May 27;55(5). doi: 10.1183/13993003.01112-2020 32312864 PMC7173682

[7] Bajgain KT, Badal S, Bajgain BB, Santana MJ. Prevalence of comorbidities among individuals with COVID-19: A rapid review of current literature. Am J Infect Control. 2021 Feb;49(2):238–246. doi: 10.1016/j.ajic.2020.06.213 32659414 PMC7351042

[8] Ji Y, Ma Z, Peppelenbosch MP, Pan Q. Potential association between COVID-19 mortality and health-care resource availability. Lancet Glob Health. 2020 Apr;8(4):e480. doi: 10.1016/S2214-109X(20)30068-1 32109372 PMC7128131

[9] Liang W, Liang H, Ou L, Chen B, Chen A, Li C, et al. Development and Validation of a Clinical Risk Score to Predict the Occurrence of Critical Illness in Hospitalized Patients With COVID-19. JAMA Intern Med. 2020 Aug 1;180(8):1081–1089. doi: 10.1001/jamainternmed.2020.2033 32396163 PMC7218676

[10] Bhargava A, Fukushima EA, Levine M, Zhao W, Tanveer F, Szpunar SM, et al. Predictors for Severe COVID-19 Infection. Clin Infect Dis. 2020 Nov 5;71(8):1962–1968. doi: 10.1093/cid/ciaa674 32472676 PMC7314166

[11] Mahdavi M, Choubdar H, Zabeh E, Rieder M, Safavi-Naeini S, Jobbagy Z, et al. A machine learning based exploration of COVID-19 mortality risk. PLoS One. 2021 Jul 2;16(7):e0252384. doi: 10.1371/journal.pone.0252384 34214101 PMC8253432

[12] Kar S, Chawla R, Haranath SP, Ramasubban S, Ramakrishnan N, Vaishya R, et al. Multivariable mortality risk prediction using machine learning for COVID-19 patients at admission (AICOVID). Sci Rep. 2021 Jun 17;11(1):12801. doi: 10.1038/s41598-021-92146-7 34140592 PMC8211710

[13] Subudhi S, Verma A, Patel AB, Hardin CC, Khandekar MJ, Lee H, et al. Comparing machine learning algorithms for predicting ICU admission and mortality in COVID-19. npj Digital Med. 2021 May 21;4(1):87. doi: 10.1038/s41746-021-00456-x 34021235 PMC8140139

[14] Magunia H, Lederer S, Verbuecheln R, Gilot BJ, Koeppen M, Haeberle HA, et al. Machine learning identifies ICU outcome predictors in a multicenter COVID-19 cohort. Crit Care. 2021 Aug 17;25(1):295. doi: 10.1186/s13054-021-03720-4 34404458 PMC8370055

[15] Hao B, Sotudian S, Wang T, Xu T, Hu Y, Gaitanidis A, et al. Early prediction of level-of-care requirements in patients with COVID-19. Elife. 2020 Oct 12;9. doi: 10.7554/eLife.60519 33044170 PMC7595731

[16] Xie J, Hungerford D, Chen H, Abrams ST, Li S, Wang G, et al. Development and external validation of a prognostic multivariable model on admission for hospitalized patients with COVID-19. medRxiv. 2020 Mar 30.

[17] Ji D, Zhang D, Xu J, Chen Z, Yang T, Zhao P, et al. Prediction for Progression Risk in Patients With COVID-19 Pneumonia: The CALL Score. Clin Infect Dis. 2020 Sep 12;71(6):1393–1399. doi: 10.1093/cid/ciaa414 32271369 PMC7184473

[18] Yan L, Zhang HT, Goncalves J, Xiao Y, Wang M, Guo Y, et al. An interpretable mortality prediction model for COVID-19 patients. Nat Mach Intell. 2020 May;2(5):283–288.

[19] Karthikeyan A, Garg A, Vinod PK, Priyakumar UD. Machine Learning Based Clinical Decision Support System for Early COVID-19 Mortality Prediction. Front Public Health. 2021 May 12;9:626697. doi: 10.3389/fpubh.2021.626697 34055710 PMC8149622

[20] Becerra-Flores M, Cardozo T. SARS-CoV-2 viral spike G614 mutation exhibits higher case fatality rate. Int J Clin Pract. 2020 Aug;74(8):e13525. doi: 10.1111/ijcp.13525 32374903 PMC7267315

[21] Saha P, Banerjee AK, Tripathi PP, Srivastava AK, Ray U. A virus that has gone viral: amino acid mutation in S protein of Indian isolate of Coronavirus COVID-19 might impact receptor binding, and thus, infectivity. Biosci Rep. 2020 May 29;40(5). 10.1042/BSR20201312 PMC7225408 32378705

[22] Wang R, Hozumi Y, Yin C, Wei G-W. Mutations on COVID-19 diagnostic targets. Genomics. 2020 Nov;112(6):5204–5213. doi: 10.1016/j.ygeno.2020.09.028 32966857 PMC7502284

[23] Korber B, Fischer WM, Gnanakaran S, Yoon H, Theiler J, Abfalterer W, et al. Tracking Changes in SARS-CoV-2 Spike: Evidence that D614G Increases Infectivity of the COVID-19 Virus. Cell. 2020 Aug 20;182(4):812–827.e19. doi: 10.1016/j.cell.2020.06.043 32697968 PMC7332439

[24] Jha IP, Awasthi R, Kumar A, Kumar V, Sethi T. Learning the Mental Health Impact of COVID-19 in the United States With Explainable Artificial Intelligence: Observational Study. JMIR Ment Health. 2021 Apr 20;8(4):e25097. doi: 10.2196/25097 33877051 PMC8059787

[25] Sethi T, Mittal A, Maheshwari S, Chugh S. Learning to Address Health Inequality in the United States with a Bayesian Decision Network. AAAI. 2019 Jul 17;33:710–717.

[26] Awasthi R, Patel P, Joshi V, Karkal S, Sethi T. Learning Explainable Interventions to Mitigate HIV Transmission in Sex Workers Across Five States in India. arXiv. 2020 Nov 30;

[27] Grasselli G, Zangrillo A, Zanella A, Antonelli M, Cabrini L, Castelli A, et al. Baseline Characteristics and Outcomes of 1591 Patients Infected With SARS-CoV-2 Admitted to ICUs of the Lombardy Region, Italy. JAMA. 2020 Apr 28;323(16):1574–1581. doi: 10.1001/jama.2020.5394 32250385 PMC7136855

[28] Xie J, Covassin N, Fan Z, Singh P, Gao W, Li G, et al. Association Between Hypoxemia and Mortality in Patients With COVID-19. Mayo Clin Proc. 2020 Jun;95(6):1138–1147. doi: 10.1016/j.mayocp.2020.04.006 32376101 PMC7151468

[29] Duan J, Wang X, Chi J, Chen H, Bai L, Hu Q, et al. Correlation between the variables collected at admission and progression to severe cases during hospitalization among patients with COVID-19 in Chongqing. J Med Virol. 2020 Nov;92(11):2616–2622. doi: 10.1002/jmv.26082 32470186 PMC7283752

[30] Yang A-P, Liu J-P, Tao W-Q, Li H-M. The diagnostic and predictive role of NLR, d-NLR and PLR in COVID-19 patients. Int Immunopharmacol. 2020 Jul;84:106504. doi: 10.1016/j.intimp.2020.106504 32304994 PMC7152924

[31] Rizo-Téllez SA, Méndez-García LA, Flores-Rebollo C, Alba-Flores F, Alcántara-Suárez R, Manjarrez-Reyna AN, et al. The Neutrophil-to-Monocyte Ratio and Lymphocyte-to-Neutrophil Ratio at Admission Predict In-Hospital Mortality in Mexican Patients with Severe SARS-CoV-2 Infection (Covid-19). Microorganisms. 2020 Oct 10;8(10). doi: 10.3390/microorganisms8101560 33050487 PMC7600553

[32] Zhao Q, Meng M, Kumar R, Wu Y, Huang J, Lian N, et al. The impact of COPD and smoking history on the severity of COVID-19: A systemic review and meta-analysis. J Med Virol. 2020 Oct;92(10):1915–1921. doi: 10.1002/jmv.25889 32293753 PMC7262275

[33] Liu W, Tao Z-W, Wang L, Yuan M-L, Liu K, Zhou L, et al. Analysis of factors associated with disease outcomes in hospitalized patients with 2019 novel coronavirus disease. Chin Med J. 2020 May 5;133(9):1032–1038. doi: 10.1097/CM9.0000000000000775 32118640 PMC7147279

[34] Mao L, Jin H, Wang M, Hu Y, Chen S, He Q, et al. Neurologic manifestations of hospitalized patients with coronavirus disease 2019 in Wuhan, China. JAMA Neurol. 2020 Apr 10. doi: 10.1001/jamaneurol.2020.1127 32275288 PMC7149362

[35] Cheng Y, Luo R, Wang K, Zhang M, Wang Z, Dong L, et al. Kidney disease is associated with in-hospital death of patients with COVID-19. Kidney Int. 2020 May;97(5):829–838. doi: 10.1016/j.kint.2020.03.005 32247631 PMC7110296

[36] Jin X, Lian J-S, Hu J-H, Gao J, Zheng L, Zhang Y-M, et al. Epidemiological, clinical and virological characteristics of 74 cases of coronavirus-infected disease 2019 (COVID-19) with gastrointestinal symptoms. Gut. 2020 Jun;69(6):1002–1009. doi: 10.1136/gutjnl-2020-320926 32213556 PMC7133387

[37] Singhal R, Rana R. Chi-square test and its application in hypothesis testing. J Pract Cardiovasc Sci. 2015;1(1):69.

[38] Chowdhury MEH, Rahman T, Khandakar A, Al-Madeed S, Zughaier SM, Doi SAR, et al. An Early Warning Tool for Predicting Mortality Risk of COVID-19 Patients Using Machine Learning. Cognit Comput. 2021 Apr 21;1–16. doi: 10.1007/s12559-020-09812-7 33897907 PMC8058759

[39] Massey FJ. The Kolmogorov-Smirnov Test for Goodness of Fit. J Am Stat Assoc. 1951 Mar;46(253):68–78.

[40] Chen T, Guestrin C. XGBoost: A Scalable Tree Boosting System. Proceedings of the 22nd ACM SIGKDD International Conference on Knowledge Discovery and Data Mining—KDD’ ’ ‘16. New York, New York, USA: ACM Press; 2016. p. 785–794.

[41] Koller D, Friedman N. Probabilistic graphical models: principles and techniques. books.google.com; 2009.

[42] R: a language and environment for statistical computing [Internet]. [cited 2021 Jul 27]. Available from: https://www.gbif.org/tool/81287/r-a-language-and-environment-for-statistical-computing.

[43] Zhao Y, Nie H-X, Hu K, Wu X-J, Zhang Y-T, Wang M-M, et al. Abnormal immunity of non-survivors with COVID-19: predictors for mortality. Infect Dis Poverty. 2020 Aug 3;9(1):108. doi: 10.1186/s40249-020-00723-1 32746940 PMC7396941

[44] Beigel JH, Tomashek KM, Dodd LE, Mehta AK, Zingman BS, Kalil AC, et al. Remdesivir for the Treatment of Covid-19—Final Report. N Engl J Med. 2020 Nov 5;383(19):1813–1826. doi: 10.1056/NEJMoa2007764 32445440 PMC7262788

[45] Olalla J. Remdesivir for the Treatment of Covid-19—Preliminary Report. N Engl J Med. 2020 Sep 3;383(10):993–994. 10.1056/NEJMc2022236 32649077

[46] Vetter P, Kaiser L, Calmy A, Agoritsas T, Huttner A. Dexamethasone and remdesivir: finding method in the COVID-19 madness. The Lancet Microbe. 2020 Dec;1(8):e309–e310. 10.1016/S2666-5247(20)30173-7 35544181

[47] RECOVERY Collaborative Group, Horby P, Lim WS, Emberson JR, Mafham M, Bell JL, et al. Dexamethasone in Hospitalized Patients with Covid-19. N Engl J Med. 2021 Feb 25;384(8):693–704. doi: 10.1056/NEJMoa2021436 32678530 PMC7383595

[48] Mitra A, Dwyre DM, Schivo M, Thompson GR, Cohen SH, Ku N, et al. Leukoerythroblastic reaction in a patient with COVID-19 infection. Am J Hematol. 2020 Aug;95(8):999–1000. doi: 10.1002/ajh.25793 32212392 PMC7228283

[49] Linssen J, Ermens A, Berrevoets M, Seghezzi M, Previtali G, van der Sar-van der Brugge S, et al. A novel haemocytometric COVID-19 prognostic score developed and validated in an observational multicentre European hospital-based study. Elife. 2020 Nov 26;9. 10.7554/eLife.63195 PMC7732342 33241996

[50] Djakpo DK, Wang Z, Zhang R, Chen X, Chen P, Antoine MMLK. Blood routine test in mild and common 2019 coronavirus (COVID-19) patients. Biosci Rep. 2020 Aug 28;40(8). doi: 10.1042/BSR20200817 32725148 PMC7414516

[51] AIX-COVNET, Roberts M, Driggs D, Thorpe M, Gilbey J, Yeung M, et al. Common pitfalls and recommendations for using machine learning to detect and prognosticate for COVID-19 using chest radiographs and CT scans. Nat Mach Intell. 2021 Mar 15.

[52] Ahmed S, Ansar Ahmed Z, Siddiqui I, Haroon Rashid N, Mansoor M, Jafri L. Evaluation of serum ferritin for prediction of severity and mortality in COVID-19- A cross sectional study. Ann Med Surg (Lond). 2021 Mar;63:102163. doi: 10.1016/j.amsu.2021.02.009 33614024 PMC7879065

