# Supplementary figures and images for: COVID-19 Risk Stratification and Mortality Prediction in Hospitalized Indian Patients: Harnessing clinical data for public health benefits
Source: PLoS One. 2022 Mar 17;17(3):e0264785. doi: 10.1371/journal.pone.0264785 (PMC8929610; doi:10.1371/journal.pone.0264785)

Figure S1: Sample Distribution for Risk Stratification with time to outcome

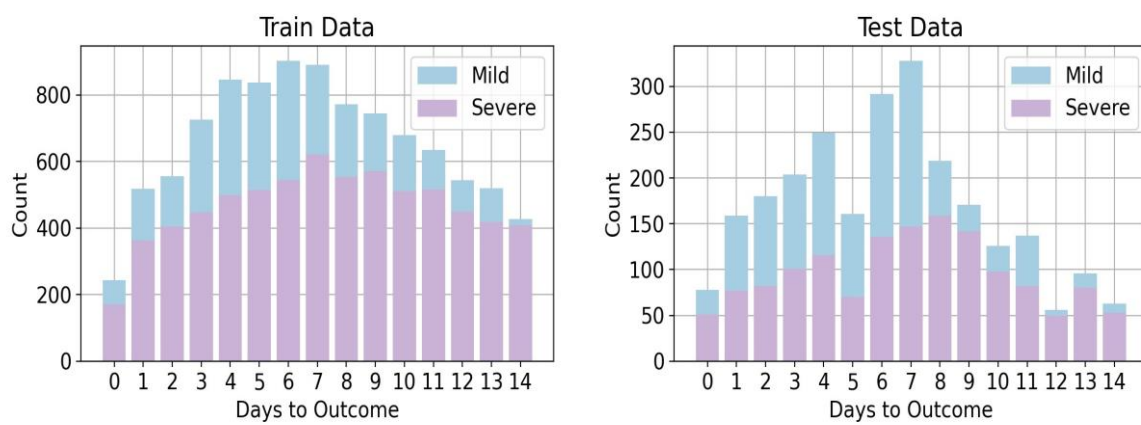

Supplement: S1 Fig — (PDF) [file pone.0264785.s001.pdf]

Figure S2: Sample Distribution for Mortality Prediction with time to outcome

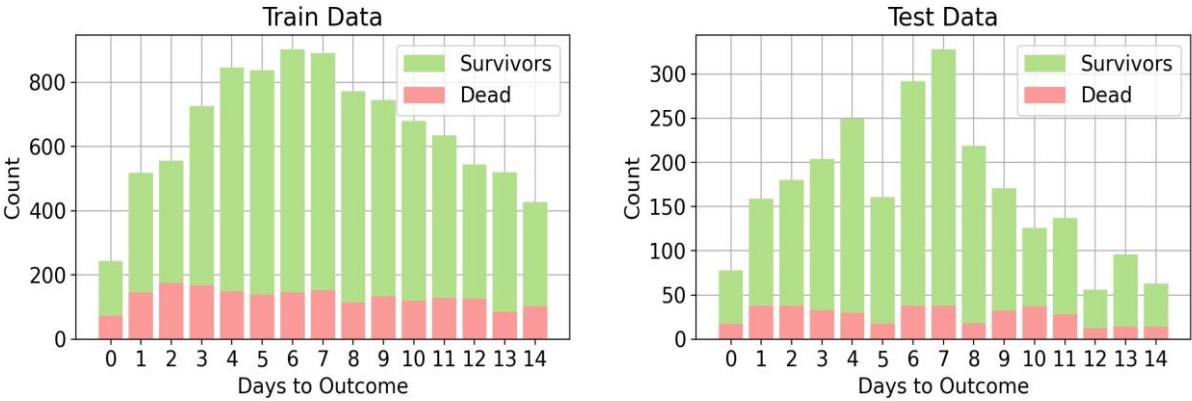

Supplement: S2 Fig — (PDF) [file pone.0264785.s002.pdf]

Figure S3: Top 25 Important Parameters for Binary Risk Stratification

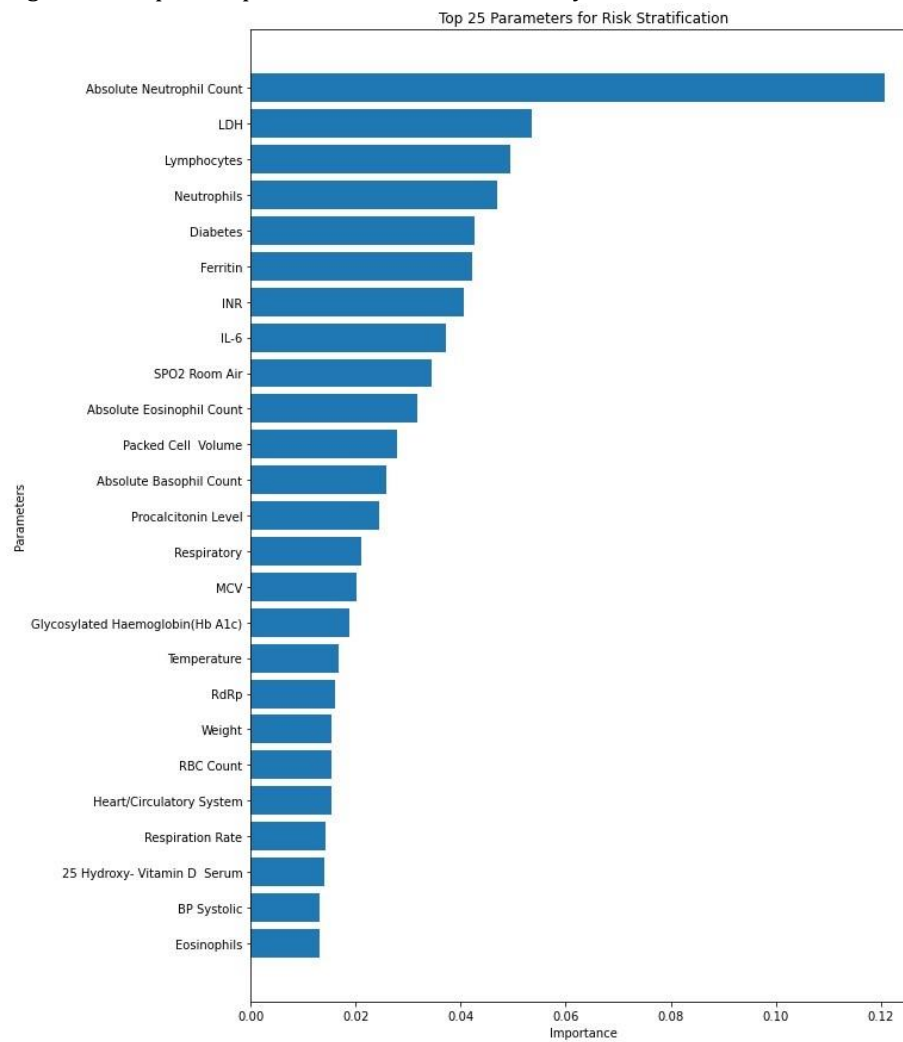

Supplement: S3 Fig — (PDF) [file pone.0264785.s003.pdf]

Figure S4: Distribution plots for four most important features used for risk stratification.

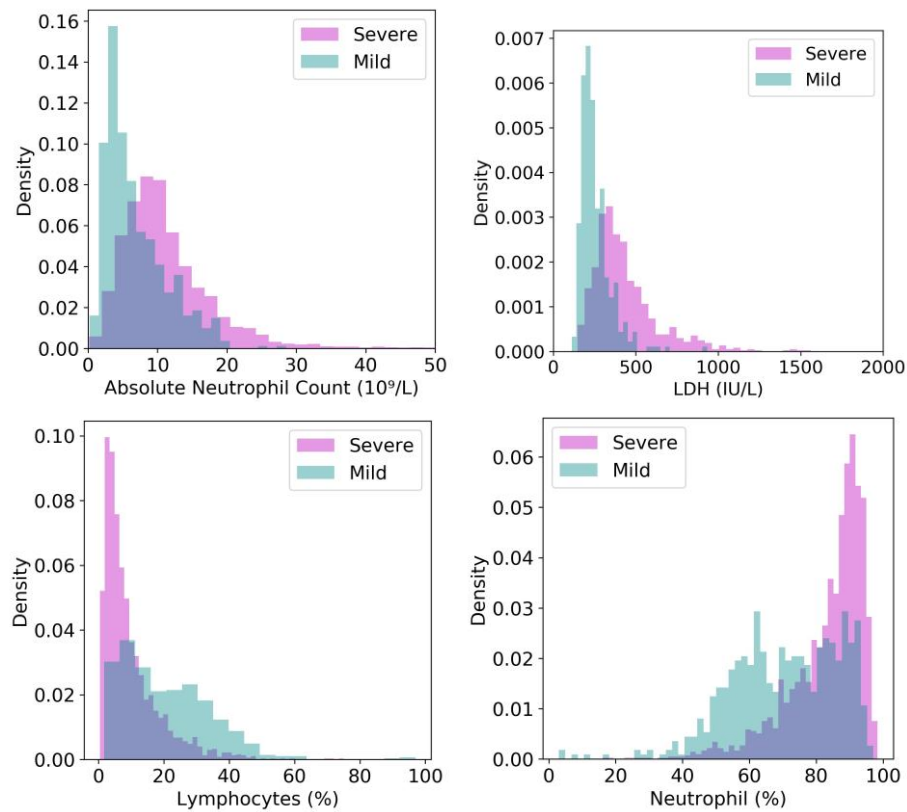

Supplement: S4 Fig — (PDF) [file pone.0264785.s004.pdf]

Figure 7: Top 25 Important Parameters for Mortality prediction

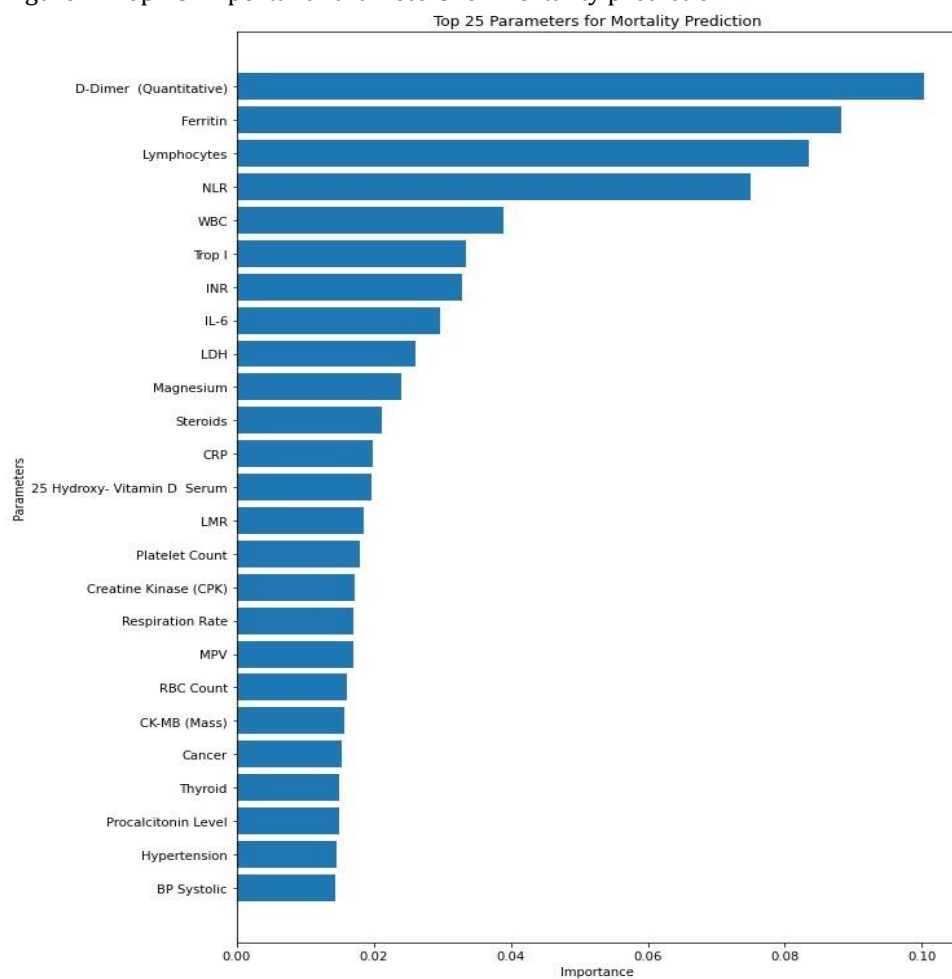

Supplement: S7 Fig — (PDF) [file pone.0264785.s007.pdf]

Figure S8: Distribution plots for four most important features used for predicting mortality.

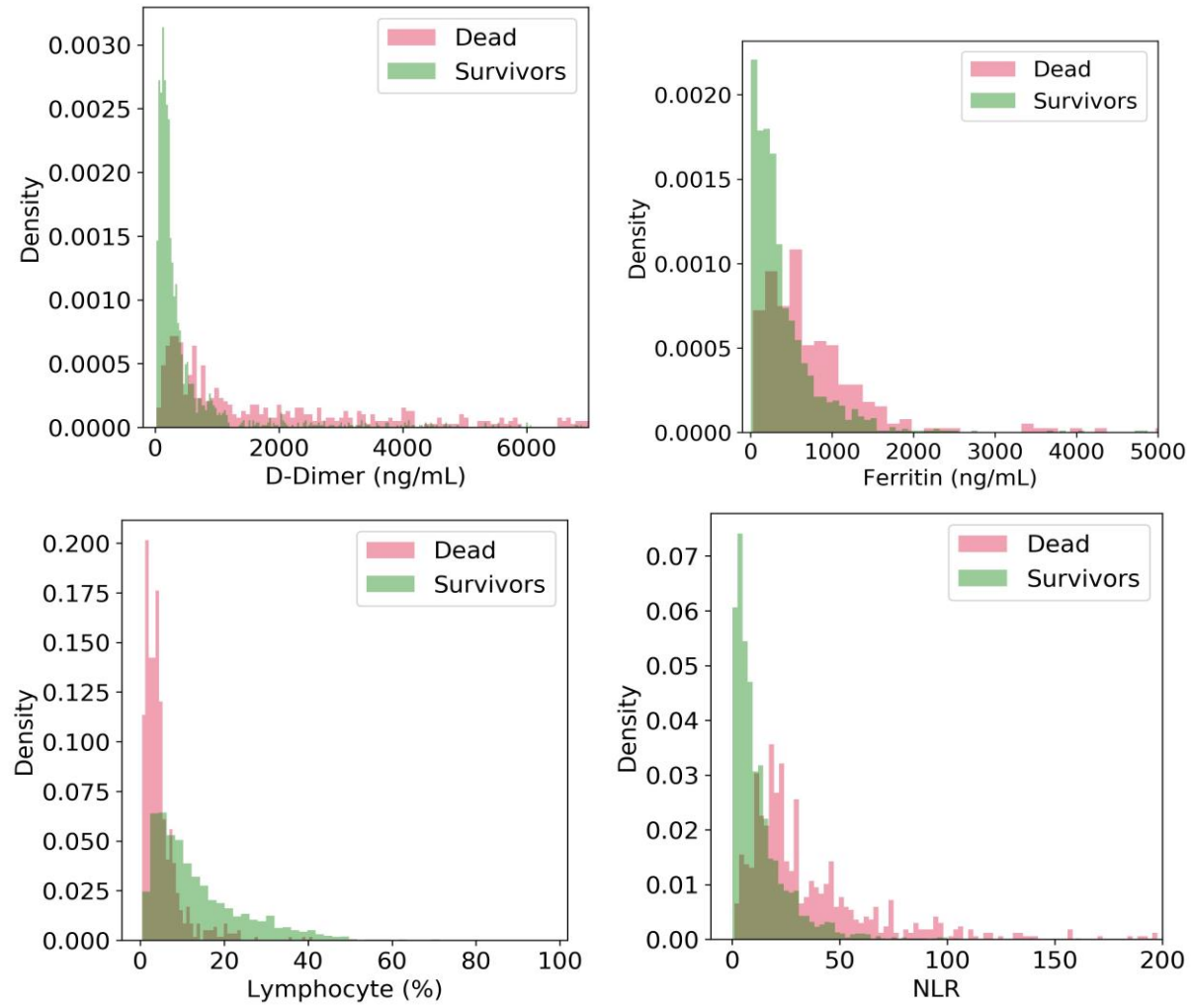

Supplement: S8 Fig — (PDF) [file pone.0264785.s008.pdf]
