# Supplementary material for: COVID-19 Risk Stratification and Mortality Prediction in Hospitalized Indian Patients: Harnessing clinical data for public health benefits
Source: PLoS One. 2022 Mar 17;17(3):e0264785. doi: 10.1371/journal.pone.0264785 (PMC8929610; doi:10.1371/journal.pone.0264785)

Figure S6: Comparison of F1 scores for various machine learning models that use only patient vitals

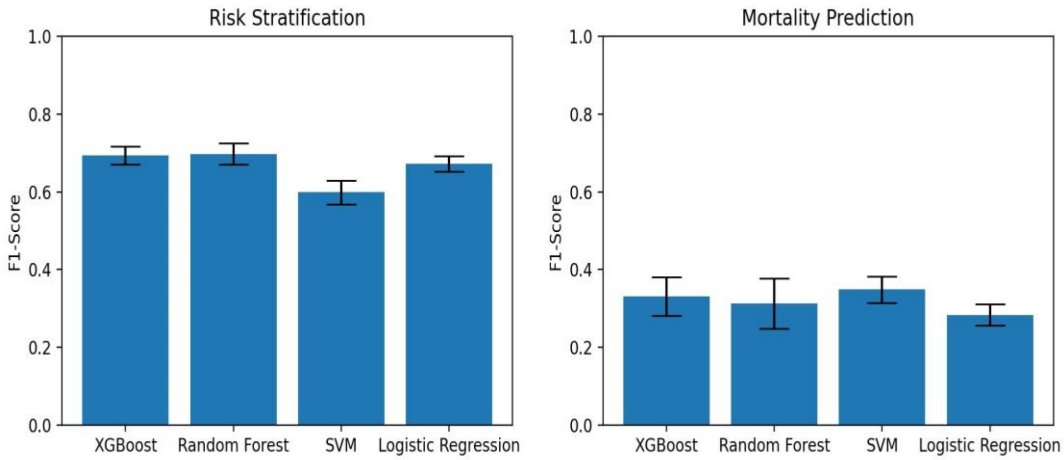

Supplement: S6 Fig — (PDF) [file pone.0264785.s006.pdf]
