# Supplementary material for: COVID-19 Risk Stratification and Mortality Prediction in Hospitalized Indian Patients: Harnessing clinical data for public health benefits
Source: PLoS One. 2022 Mar 17;17(3):e0264785. doi: 10.1371/journal.pone.0264785 (PMC8929610; doi:10.1371/journal.pone.0264785)
Supplement: S1 Table — Medians and P-values are given for individual features. (PDF) [file pone.0264785.s009.pdf]

Table S1: Categorical features for Risk Stratification. Medians and P-values are given for individual features.

**Statistical Analysis for Categorical Features**			
	**Risk Stratification**		
**Feature Name**	**High Risk [n (%)]**	**Low Risk [n (%)]**	**P-value**
Sex, Female	78 (26.0)	86 (28.67)	.05
Pregnancy	0 (0.0)	1 (0.33)	.27
Steroids prescribed	225 (75.0)	113 (37.67)	$\ll .001$
AntiVirals prescribed	207 (69.0)	181 (60.33)	.48
Hypertension	159 (53.0)	89 (29.67)	.005
Diabetes	147 (49.0)	81 (27.0)	.005
Cancer	7 (2.33)	7 (2.33)	.70
Hyperlipidemia/Dislipidemia	10 (3.33)	5 (1.67)	.37
Thyroid related illness	42 (14.0)	44 (14.67)	.24
Heart/Circulatory System related illness	47 (15.67)	18 (6.0)	.005
Respiratory illness	31 (10.33)	24 (8.0)	0.856
Brain/Nervous System illness	16 (5.33)	8 (2.67)	.26
Renal illness	28 (9.33)	16 (5.33)	.26
Liver related illness	5 (1.67)	4 (1.33)	.98
presence of any Other illness	60 (20.0)	29 (9.67)	.02
