# Supplementary material for: COVID-19 Risk Stratification and Mortality Prediction in Hospitalized Indian Patients: Harnessing clinical data for public health benefits
Source: PLoS One. 2022 Mar 17;17(3):e0264785. doi: 10.1371/journal.pone.0264785 (PMC8929610; doi:10.1371/journal.pone.0264785)
Supplement: S2 Table — Medians and P-values are given for individual features. (PDF) [file pone.0264785.s010.pdf]

Table S2: Continuous features for Risk Stratification. Medians and P-values are given for individual features.

Statistical Analysis (Numerical Features)			
**Feature Name**	**Risk Stratification**		
	**High Risk (IQR)**	**Low Risk (IQR)**	**P-value**
Glycosylated Haemoglobin (Hb A1c) (%)	6.7 (5.94-7.7)	6.1 (5.54-7.19)	$\ll .001$
Average Glucose Value For the Last 3 Months (mg/dl)	145.5 (123.7-174.2)	128.3 (112.3-159.6)	$\ll .001$
Average Glucose Value For the Last 3 Months IFCC (mmol/L)	8.06 (6.86-9.65)	7.11 (6.22-8.84)	$\ll .001$
Glycosylated Haemoglobin (Hb A1c) IFCC (mmol/mol)	49.72 (41.41-60.65)	43.16 (37.04-55.07)	$\ll .001$
Temperature ($^{\circ}$F)	98.2 (97.8-98.6)	98.0 (97.3-98.6)	.36
BP Systolic (mmHg)	130.0 (120.0-140.0)	130.0 (120.0-139.0)	$\ll .001$
BP Diastolic (mmHg)	80.0 (70.0-84.0)	80.0 (70.0-82.0)	$\ll .001$
Pulse Rate	88.0 (80.0-102.0)	86.0 (79.0-96.0)	$\ll .001$
SPO_2 (Room Air)	94.0 (88.0-97.0)	97.0 (96.0-98.0)	$\ll .001$

Respiration Rate	20.0 (20.0-24.0)	20.0 (20.0-22.0)	$\ll$ .001
25 Hydroxy- Vitamin D Serum (ng/mL)	26.25 (12.94-41.76)	22.72 (10.56-38.94)	.12
Basophils (%)	0.3 (0.1-0.5)	0.3 (0.12-0.5)	.87
Eosinophils (%)	0.1 (0.0-0.5)	0.3 (0.0-1.3)	$\ll$ .001
Haemoglobin (g/dl)	12.2 (10.5-13.5)	12.8 (11.0-14.0)	$\ll$ .001
Lymphocytes (%)	6.6 (3.6-12.5)	18.05 (9.12-29.87)	$\ll$ .001
MCH (pg)	28.7 (26.6-30.2)	28.9 (27.2-30.5)	.002
MCHC (g/dl)	33.1 (32.2-34.0)	33.4 (32.6-34.1)	$\ll$ .001
MCV (fL)	86.2 (81.38-90.12)	86.4 (82.5-89.98)	.03
Monocytes (%)	5.1 (3.4-7.8)	8.0 (5.22-9.9)	$\ll$ .001
Neutrophils (%)	87.2 (78.35-91.4)	71.75 (59.12-84.1)	$\ll$ .001
Packed Cell Volume (%)	36.9 (32.1-40.55)	38.1 (33.32-41.48)	$\ll$ .001
Platelet Count (10^9/L)	231.0 (165.0-311.0)	220.5 (170.0-292.0)	.019
RDW (%)	15.1 (14.1-16.5)	14.6 (13.8-15.6)	$\ll$ .001
Total Leucocyte Count (TLC) (10^9/L)	11.6 (8.7-15.85)	7.8 (5.7-11.4)	$\ll$ .001
Absolute Lymphocyte Count (10^9/L)	0.78 (0.47-1.3)	1.36 (0.86-1.92)	$\ll$ .001
Absolute Neutrophil Count (10^9/L)	9.82 (6.92-13.97)	5.51 (3.4-9.25)	$\ll$ .001
Absolute Monocyte Count (10^9/L)	0.58 (0.39-0.86)	0.55 (0.4-0.76)	.008
RBC Count (10^{12}/L)	4.36 (3.85-4.76)	4.43 (3.99-4.79)	.02
MPV (fL)	9.1 (8.4-10.2)	9.2 (8.4-10.68)	.01
WBC	11.59 (8.69-15.85)	7.8 (5.7-11.4)	$\ll$ .001
NLR	13.19 (6.33-25.24)	3.94 (1.99-8.82)	$\ll$ .001
LMR	1.39 (0.84-2.15)	2.42 (1.56-3.56)	$\ll$ .001
NMR	16.59 (9.99-26.95)	8.54 (6.3-15.39)	$\ll$ .001
PLR	300.0 (177.7-525.0)	169.9 (109.6-268.1)	$\ll$ .001
CRP (mg/L)	23.66 (6.37-72.56)	8.99 (2.99-32.71)	$\ll$ .001
Absolute Basophil Count (10^9/L)	0.04 (0.02-0.07)	0.03 (0.02-0.05)	$\ll$ .001
Absolute Eosinophil Count (10^9/L)	0.05 (0.02-0.12)	0.06 (0.02-0.12)	.35
Ferritin (ng/mL)	387.2 (211.0-706.2)	147.4 (66.1-302.0)	$\ll$ .001
Trop I (ng/mL)	0.01 (0.01-0.03)	0.01 (0.0-0.01)	.12
Procalcitonin Level (ng/mL)	0.11 (0.07-0.26)	0.05 (0.03-0.1)	.004
CK-MB (Mass) (ng/mL)	2.0 (1.0-4.7)	1.1 (0.7-1.9)	.02
IL-6 (pg/ml)	35.94 (9.73-122.25)	7.84 (3.25-22.43)	$\ll$ .001
INR	1.13 (1.04-1.27)	1.05 (0.99-1.16)	.06
Prothrombin Time (sec)	12.8 (11.8-14.4)	12.0 (11.2-13.1)	.06
D-Dimer (Quantitative) (ng/mL)	384.0 (188.0-1122.9)	143.5 (81.3-239.9)	$\ll$ .001
Magnesium (mg/dl)	2.1 (1.9-2.3)	2.0 (1.9-2.2)	.02
LDH (IU/L)	384.5 (301.0-508.2)	231.0 (193.0-292.0)	$\ll$ .001

Creatine Kinase (U/L)	106.0 (52.5-209.25)	86.5 (60.75-154.25)	.05
E (%)	24.76 (21.38-27.9)	24.13 (20.83-27.07)	.2
RdRp (mg/dl)	27.15 (22.2-29.36)	25.35 (22.82-28.16)	0.56
Age (yrs)	61.0 (53.0-68.0)	53.0 (41.0-64.0)	$\ll .001$
Height (cms)	162.0 (157.0-170.0)	165.0 (158.0-171.2)	.86
Weight (kg)	73.15 (63.08-82.0)	72.5 (63.0-80.0)	.45
BMI	26.32 (23.89-30.11)	26.31 (24.05-28.13)	.11
