# Supplementary material for: COVID-19 Risk Stratification and Mortality Prediction in Hospitalized Indian Patients: Harnessing clinical data for public health benefits
Source: PLoS One. 2022 Mar 17;17(3):e0264785. doi: 10.1371/journal.pone.0264785 (PMC8929610; doi:10.1371/journal.pone.0264785)
Supplement: S3 Table — Medians and P-values are given for individual features. (PDF) [file pone.0264785.s011.pdf]

Table S3: Categorical features for Mortality Prediction. Medians and P-values are given for individual features.

Statistical Analysis for Categorical Features			
**Feature Name**	**Risk Stratification**		
	**Died [n (%)]**	**Survived [n (%)]**	**P-value**
Sex, Female	16 (26.23)	148 (30.64)	.55
Pregnancy	0 (0.0)	1 (0.21)	.72
Steroids prescribed	35 (57.38)	303 (62.73)	.62
AntiVirals prescribed	31 (50.82)	357 (73.91)	.04
Hypertension	40 (65.57)	208 (43.06)	.01
Diabetes	34 (55.74)	194 (40.17)	.08
Cancer	3 (4.92)	11 (2.28)	.23
Hyperlipidemia/Dislipidemia	0 (0.00)	15 (3.11)	.17
Thyroid related illness	10 (16.39)	76 (15.73)	.9
Heart/Circulatory System related illness	14 (22.95)	51 (10.56)	.01
Respiratory illness	7 (11.48)	48 (9.94)	.72
Brain/Nervous System illness	3 (4.92)	21 (4.35)	.84
Renal illness	12.0 (19.67)	32.0 (6.63)	$\ll .001$
Liver related illness	3.0 (4.92)	6.0 (1.24)	.04
presence of any Other illness	15.0 (24.59)	74.0 (15.32)	.09
