# Supplementary material for: COVID-19 Risk Stratification and Mortality Prediction in Hospitalized Indian Patients: Harnessing clinical data for public health benefits
Source: PLoS One. 2022 Mar 17;17(3):e0264785. doi: 10.1371/journal.pone.0264785 (PMC8929610; doi:10.1371/journal.pone.0264785)
Supplement: S4 Table — Medians and P-values are given for individual features. (PDF) [file pone.0264785.s012.pdf]

Table S4: Continuous features for Mortality Prediction. Medians and P-values are given for individual features.

Statistical Analysis (Numerical Features)			
**Feature Name**	**Risk Stratification**		
	**High Risk (IQR)**	**Low Risk (IQR)**	**P-value**
Glycosylated Haemoglobin(Hb A1c) (%)	6.17 (5.76-7.12)	6.38 (5.7-7.52)	.40
Average Glucose Value For the Last 3 Months (mg/dl)	130.38 (118.61-157.5)	136.26 (116.96-169.05)	.40

Average Glucose Value For the Last 3 Months IFCC (mmol/L)	7.22 (6.57-8.72)	7.54 (6.48-9.36)	.40
Glycosylated Haemoglobin(Hb A1c) IFCC (mmol/mol)	43.92 (39.44-54.25)	46.16 (38.81-58.65)	.40
Temperature (˚F)	98.2 (98.0-98.6)	98.1 (97.5-98.6)	.004
BP Systolic (mmHg)	130.0 (124.0-140.0)	130.0 (120.0-140.0)	.09
BP Diastolic (mmHg)	80.0 (70.0-80.0)	80.0 (70.0-84.0)	$\ll$ .001
Pulse Rate	88.0 (80.0-107.0)	88.0 (80.0-100.0)	$\ll$ .001
SPO_2 (Room Air)	92.0 (80.0-96.0)	96.0 (93.0-98.0)	$\ll$ .001
Respiration Rate	22.0 (20.0-26.0)	20.0 (20.0-22.0)	$\ll$ .001
25 Hydroxy- Vitamin D Serum (ng/mL)	26.06 (14.52-39.53)	24.1 (11.07-40.64)	.49
Basophils (%)	0.2 (0.1-0.4)	0.3 (0.2-0.6)	$\ll$ .001
Eosinophils (%)	0.1 (0.0-0.3)	0.1 (0.0-0.8)	$\ll$ .001
Haemoglobin (g/dl)	11.5 (9.7-13.3)	12.5 (10.9-13.7)	$\ll$ .001
Lymphocytes (%)	3.8 (2.0-5.6)	10.1 (5.55-18.8)	$\ll$ .001
MCH (pg)	28.7 (26.7-30.2)	28.7 (26.8-30.3)	0.41
MCHC (g/dl)	32.8 (31.8-33.7)	33.3 (32.4-34.1)	$\ll$ .001
MCV (fL)	87.0 (82.18-90.7)	86.1 (81.4-90.0)	$\ll$ .001
Monocytes (%)	3.9 (2.6-5.9)	6.3 (4.0-8.9)	$\ll$ .001
Neutrophils (%)	91.0 (87.45-93.8)	82.4 (70.8-89.2)	$\ll$ .001
Packed Cell Volume (%)	35.4 (30.05-39.4)	37.5 (33.1-41.0)	$\ll$ .001
Platelet Count (10^9/L)	185.0 (155.0-265.5)	241.0 (174.0-319.0)	$\ll$ .001
RDW (%)	15.3 (14.4-16.7)	14.9 (14.0-16.2)	$\ll$ .001
Total Leucocyte Count (TLC) (10^9/L)	15.5 (10.9-20.8)	10.2 (7.3-13.3)	$\ll$ .001
Absolute Lymphocyte Count (10^9/L)	0.56 (0.31-0.9)	1.0 (0.6-1.64)	.05
Absolute Neutrophil Count (10^9/L)	13.99 (9.62-18.9)	8.19 (5.24-11.29)	$\ll$ .001
Absolute Monocyte Count (10^9/L)	0.56 (0.37-0.91)	0.58 (0.4-0.83)	$\ll$ .001
RBC Count (10^{12}/L)	4.16 (3.5-4.66)	4.43 (3.97-4.79)	$\ll$ .001
MPV (fL)	9.6 (8.65-10.5)	9.0 (8.3-10.1)	$\ll$ .001
WBC	15.5 (10.9-20.8)	10.2 (7.3-13.3)	$\ll$ .001
NLR	23.97 (15.65-45.44)	8.15 (3.78-15.95)	$\ll$ .001
LMR	0.9 (0.55-1.52)	1.73 (1.14-2.67)	$\ll$ .001
NMR	23.57 (14.97-35.83)	12.72 (7.93-21.85)	$\ll$ .001
PLR	385.71 (207.61-691.49)	242.73 (145.91-419.21)	$\ll$ .001
CRP (mg/L)	59.01 (18.33-122.56)	14.7 (4.3-50.36)	$\ll$ .001
Absolute Basophil Count (10^9/L)	0.04 (0.02-0.08)	0.03 (0.02-0.06)	$\ll$ .001

Absolute Eosinophil Count (10^9/L)	0.04 (0.02-0.12)	0.05 (0.02-0.12)	.02
Ferritin (ng/mL)	603.9 (327.75-1052.4)	260.0 (119.85-505.65)	$\ll .001$
Trop I (ng/mL)	0.02 (0.01-0.14)	0.01 (0.0-0.01)	$\ll .001$
Procalcitonin Level (ng/mL)	0.18 (0.09-0.67)	0.08 (0.05-0.16)	.005
CK-MB (Mass) (ng/mL)	3.9 (1.6-8.3)	1.3 (0.8-2.7)	$\ll .001$
IL-6 (pg/ml)	157.1 (39.27-626.9)	16.13 (4.94-51.61)	$\ll .001$
INR	1.26 (1.14-1.52)	1.1 (1.02-1.19)	$\ll .001$
Prothrombin Time (sec)	14.3 (12.95-17.3)	12.4 (11.5-13.55)	$\ll .001$
D-Dimer (Quantitative) (ng/mL)	1386.5 (488.75-3987.55)	219.0 (126.5-454.5)	$\ll .001$
Magnesium (mg/dl)	2.1 (1.9-2.48)	2.1 (1.9-2.2)	.05
LDH (IU/L)	527.0 (404.0-743.0)	310.0 (233.0-400.25)	$\ll .001$
Creatine Kinase (U/L)	123.0 (58.0-282.5)	89.0 (57.0-170.0)	0.11
E (%)	22.94 (15.72-27.69)	24.76 (21.48-27.67)	$\ll .001$
RdRp (mg/dl)	26.98 (18.24-28.45)	26.5 (22.82-29.36)	$\ll .001$
Age (years)	65.5 (60.75-70.25)	57.0 (46.0-65.5)	$\ll .001$
Height (cms)	160.2 (159.5-167.0)	165.0 (157.0-170.0)	.92
Weight (kg)	65.1 (61.45-74.15)	73.0 (63.0-82.0)	.26
BMI	24.08 (23.25-25.75)	26.63 (24.04-28.8)	.16
