# Supplementary material for: COVID-19 Risk Stratification and Mortality Prediction in Hospitalized Indian Patients: Harnessing clinical data for public health benefits
Source: PLoS One. 2022 Mar 17;17(3):e0264785. doi: 10.1371/journal.pone.0264785 (PMC8929610; doi:10.1371/journal.pone.0264785)
Supplement: S5 Table — (PDF) [file pone.0264785.s013.pdf]

Table S5: Performance of the developed machine learning algorithms in risk stratification reported as mean ± standard deviation.

Risk Stratification			
**Algorithm**	**AUC**	**F1 score**	**Average Precision**
XGBoost	0.833 ± 0.01	0.810 ± 0.01	0.891 ± 0.01
Random forest	0.826 ± 0.01	0.803 ± 0.01	0.878 ± 0.01
SVM	0.817 ± 0.01	0.752 ± 0.01	0.889 ± 0.01
Logistic regression	0.812 ± 0.01	0.759 ± 0.01	0.885 ± 0.01
