# Supplementary material for: COVID-19 Risk Stratification and Mortality Prediction in Hospitalized Indian Patients: Harnessing clinical data for public health benefits
Source: PLoS One. 2022 Mar 17;17(3):e0264785. doi: 10.1371/journal.pone.0264785 (PMC8929610; doi:10.1371/journal.pone.0264785)
Supplement: S6 Table — (PDF) [file pone.0264785.s014.pdf]

Table S6: Performance of the developed machine learning algorithms in mortality prediction reported as mean ± standard deviation.

**Mortality Prediction**			
**Algorithm**	**AUC**	**F1 score**	**Average Precision**
XGBoost	0.891 ± 0.02	0.634 ± 0.02	0.732 ± 0.03
Random forest	0.858 ± 0.01	0.555 ± 0.03	0.615 ± 0.03
SVM	0.895 ± 0.01	0.659 ± 0.02	0.710 ± 0.02
Logistic regression	0.927 ± 0.01	0.710 ± 0.02	0.801 ± 0.02
