# Supplementary material for: COVID-19 Risk Stratification and Mortality Prediction in Hospitalized Indian Patients: Harnessing clinical data for public health benefits
Source: PLoS One. 2022 Mar 17;17(3):e0264785. doi: 10.1371/journal.pone.0264785 (PMC8929610; doi:10.1371/journal.pone.0264785)
Supplement: S7 Table — (PDF) [file pone.0264785.s015.pdf]

Table S7: The inferences using the Exact Inference algorithm on the learned structure.

**Variable 1 (Outcome Inferred)**	**Variable 2 (Conditioned upon)**	**Percent Bootstrapped networks having the association**	**% Difference in Variable 1 Inferred by setting low and high intervals of Variable 2 (Direction of correlation)**
Mortality	Severity	100%	100% (positive correlation)
Mortality	Ferritin	82%	55% (positive correlation)
Days to Outcome (Death within 7 days)	Serum levels of Vitamin D	55%	6% (positive correlation)
Days to Outcome (Death within 7 days)	Severity class	100%	34% (negative correlation)
Days to Outcome (Death within 7 days)	Pulse rate	55%	3% (positive correlation)
Days to Outcome (Death within 7 days)	Platelet count	100%	2% (positive correlation)
Days to Outcome (Death within 7 days)	RBC Count	82%	7% (positive correlation)
